# The Complex Valorization of Black Alder Bark Biomass in Compositions of Rigid Polyurethane Foam

**DOI:** 10.3390/ma18010050

**Published:** 2024-12-26

**Authors:** Alexandr Arshanitsa, Matiss Pals, Laima Vevere, Lilija Jashina, Oskars Bikovens

**Affiliations:** Latvian State Institute of Wood Chemistry, Dzerbenes Street 27, LV-1006 Riga, Latvia; matiss.pals@kki.lv (M.P.); laima.vevere@kki.lv (L.V.); lilija_jasina@inbox.lv (L.J.); oskars.bikovens@kki.lv (O.B.)

**Keywords:** black alder bark, extractives, bio-polyol, insulating material, rigid polyurethane foam

## Abstract

The use of black alder (BA) bark biomass in rigid polyurethane (PUR) foam compositions was the main task of investigation. Extractive compounds isolated from the bark through hot water extraction were used as precursors for bio-polyol synthesis via acid-free liquefaction with the polyether polyol Lupranol 3300 and through oxypropylation with propylene carbonate. The OH functionality and composition of the polyols were analyzed via wet chemistry and FTIR spectroscopy. The solid remaining after the isolation of extractive compounds was also utilized as a natural filler in PUR foams. The effects of replacing commercial polyols with bio-polyols on the foam rising rate and their mechanical properties, morphology, thermal conductivity, and thermal degradation characteristics were examined. The oxypropylated extractive-based PUR compositions demonstrated the most favorable balance between the biomass content and material properties. At an apparent density of 40 kg/m^3^, the compressive strength of the produced foams was enhanced by 1.4–1.5 times, while the maximum thermal degradation rate in air decreased by 3.8–6.5 times compared to reference materials without adversely affecting the foam morphology. The composition based on liquefied extractives showed lower performance but still improved properties relative to the reference foams. Introducing 3.7–14% of extracted bark into the foam compositions increased the biomass content to 22–24%, although this led to a decrease in the compressive strength and thermal stability. It was shown that partially substituting fossil-derived components with renewable bark biomass in the composition of PUR foams allows for materials with characteristics similar or better to petrochemical-based materials to be obtained. Therefore, the results presented can be considered a contribution to addressing environmental problems and promoting the development of a sustainable economy.

## 1. Introduction

Today, rigid polyurethane (PUR) foams are extensively used as an engineering material due to their versatility and exceptional performance, combining low density with high thermal insulation and mechanical properties. The rapid growth of the building and construction industry has been a major driver for the demand for PUR foam insulation in buildings to reduce energy consumption, lower overall infrastructure costs, and meet sustainability requirements. The refrigerator/freezer and automotive industries are other important sectors that widely use PUR foam as an effective thermal insulation and energy-absorbing material, respectively. The significance of this material for modern industry development is confirmed by statistical data. The global rigid PUR foam market size was valued at USD 20.7 million in 2023 and is expected to grow at an annual rate of 5.8% from 2023 to 2030 [1].

Diphenylmethane diisocyanate (MDI), its derivatives, and most polyols, which serve as the building blocks for rigid PUR foam production, are currently derived from fossil resources [2,3]. Environmental concerns and the depletion of fossil resources are driving the development of strategies to decrease petrochemical components in polymer compositions, including PUR foam, by partially substituting them with renewable and sustainable macromonomers. This approach aligns with the Paris Agreement, signed by 196 countries, which aims to reduce greenhouse gas emissions by 80% by 2050 compared to 1990 levels, making the world climate-neutral [4]. Industrially, MDI is produced by the interaction of phosgene with methylenedianiline. The MDI global market is estimated at 8.09 million tons and is expected to reach 10.46 million tons by 2029 [5]. Phosgene is hazardous to human health, and the process is not environmentally friendly. However, there is currently no viable greener, phosgene-free industrial technology for isocyanate production, indicating a need for further research and development [6]. Consequently, the most attractive approach for today’s industry to produce bio-based polyurethane is the partial or complete substitution of fossil-based polyols with renewable alternatives while retaining fossil-derived polyisocyanates in the PUR compositions [7]. Liquid vegetable oils isolated from plant seeds are sustainable, bio-derived, and environmentally friendly resources. These oils have been actively tested as precursors for polyols in the production of PUR foam [8]. Castor oil [9] and various edible oils, including soybean [10], linseed [11], olive [12], rapeseed [13], and sunflower [14], have also been explored for these purposes.

The transesterification/transamination of Castrol and other vegetable oils with polyhydroxy alcohols, such as glycerol, diethylene glycol, pentaerythritol, and di- and triethanolamine, is a common pathway to introduce hydroxyl functionality into vegetable oil-based polyols [8,15,16,17].

Another approach involves the epoxidation of the double bonds present in triglycerides, followed by oxirane ring-opening reactions with polyfunctional alcohols, such as trimethylolpropane, diethylene glycol, and di- and tri-ethanol amines [13,14]. Similar pathways have been tested for tall oil, including crude oil, a byproduct of the Kraft pulping process [18,19].

Lignocellulosic materials, such as cereal crops and wood biomass, including processing waste, represent another cheap and sustainable resource for the PUR industry. The major components of lignocellulosic biomass—cellulose, hemicellulose, and extractives—differ significantly in chemical structure. However, unlike vegetable oils, all of these components are rich in hydroxyl groups, which allows lignocellulosic biomass to be characterized as a natural polyol [20].

Importantly, lignocellulosic biomass does not compete with the food market, which is considered a significant advantage over renewable oils [21]. However, unlike vegetable oils, lignocellulosic biomass is in a solid state. Therefore, its transformation into a liquid form is necessary for use as polyols in PUR foam compositions [22].

Effective methods for the liquefaction of lignocellulosic biomass via the interaction of OH groups in the biomass with propylene oxide (PO) through an anionic polymerization mechanism catalyzed by alkali were developed by Glasser [23,24]. Oxypropylated lignin was first carefully examined as a lignocellulosic-based polyol for use in PUR foam compositions, replacing fossil-based polyols [25].

Subsequently, this method was successfully adapted for the synthesis of bio-polyols using cork and tree bark [26,27], rapeseed cake residue [28], and sugar beet [29], all of which are suitable for rigid PUF foam compositions. The short-chain grafting of PO onto OH groups in biomass constituents reduced electronic and steric constraints, thereby increasing their reactivity with isocyanates [30].

However, the low explosive limit, high flammability, and carcinogenicity of PO are significant disadvantages of this method. In this context, oxyalkylation with propylene carbonate (PC) is considered a much more environmentally friendly alternative to the conventional oxypropylation of biomass [31]. The process operates at 100–170 °C and does not require high-pressure equipment [2,32]. The dominance of the oxypropylation mechanism over transcarbonation during PC ring opening results in the formation of structures similar to those formed in the conventional oxypropylation of lignin and bark extractives [2,33].

The liquefaction (glycolysis) of various biomass materials, including bamboo [34], wheat straw [35], lignin [2,36], and bark [37,38], using polyhydric alcohols, such as glycerol, polyethylene glycols, or polypropylene glycol of varying molecular weights—or their combinations—in the presence of sulfuric acid as a catalyst at 110–180 °C, has proven to be an effective method for obtaining low-viscosity polyols suitable for PUR processing.

A mixture of PEG 400 (Mn = 400 g·mol^−1^) with 10–15% glycerol is most commonly used to produce lignocellulosic-based polyols suitable for PUR foam processing. The addition of glycerol prevents recondensation reactions, thereby increasing the yield of liquefaction [39]. Nevertheless, the biomass origin significantly influences the liquefaction yield, biomass content in the final polyol, and the effect of substituting fossil-based polyols on the properties of the resulting PUR foam. For example, the liquefaction yield of nutshells from different species of the Camellia family varied in the range of 50–80%, resulting in a biomass content in polyols of 12–20% [40].

At similar conditions of walnut shell liquefaction, only 8% of the biomass content in polyol was achieved. Nevertheless, the small content of liquified biomass in the composition, a significant increase in compression characteristics, and a decrease in the water uptake for PUR foam was reported [21].

It is recognized that among different lignocellulosic raw materials, tree bark—underutilized renewable biomass with an annual production of 300–400 million m^3^, which is often incinerated or landfilled without valorizing its content—has significant potential for the development of different composite materials, including particle boards, plywood, and polyurethanes [38,41,42]. However, the data on the impact of polyols derived from bark on the properties of PUR foam are ambiguous and are predominantly associated with bark from the pine family. Through the liquefaction of bark from *Pinus contorta*, polyols with a biomass content of 8–23% were synthesized. It was found that short-chain, primary hydroxyl-bearing polyhydric alcohols used as liquefaction agents revealed improved bark conversion, and the resulting polyols, in terms of the hydroxyl value (OHV) and viscosity, were suitable for rigid PUR foam compositions. In contrast, polyols based on higher equivalent alcohols would be better suited for flexible PUR foams [38].

On the other hand, the liquefaction of pine bark using a mixture of PEG 400 (90%) and glycerol (10%) in the presence of xylene as a co-solvent resulted in the synthesis of polyols with a biomass content of 12–14%. The introduction of this polyol into the PUR foam composition caused a high open-cell content in the foam and a drastic decrease in its Young’s modulus under compression [37]. Substituting the liquefied bark polyol with an oxypropylated bark polyol resulted in a significant increase in the compression characteristics of PUR foam [26].

Black alder (*Alnus glutinosa*) is a widespread, fast-growing, and adaptive tree species in Europe, which has a big potential for the furniture industry [43].

Earlier authors have shown that the bark of black alder is a source of hydrophilic extractives, including diarylheptanoids, flavonoids in monomeric and oligomeric form, carbohydrates, lignin-related phenols, organic acid, etc., which can be easily isolated via the fast and low energy expensive microwave-assisted extraction of bark with water at 70–90 °C [44].

The high concentration of OH groups of different origins in BA bark extracts allows us to consider them available precursors for synthesizing bio-polyols suitable for applications in rigid PUR foam compositions. In this work, the effects of bio-polyols obtained via both the “green” oxypropylation of BA bark extract with PC and by its liquefaction with the commercial polyether polyol Lupranol 3300 on the foaming process and properties of synthesized rigid PUR foam were studied. The bark remaining after extraction was introduced as a filler to evaluate the proportion of biomass in the PUR foam without compromising the main morphological, mechanical, and thermal characteristics of the material compared to a reference system based on commercial polyols.

## 2. Materials and Methods

### 2.1. Materials for Synthesis of Bio-Polyols and PUR Foams

BA was harvested from approximately 27-year-old trees grown in the Talsi municipality of Latvia. The extract isolated from BA bark via microwave-assisted extraction with hot (90 °C) water, as described in [44,45], was used as a renewable precursor for bio-polyol processing.

Two bio-polyols with hydroxyl values (OHV) of 464 and 538 mg KOH∙g^−1^ were synthesized by the authors via two pathways: liquefaction and “oxypropylation” of extractives isolated through the microwave-assisted extraction of BA bark, respectively. The commercial oxypropylated glycerol-based polyol-polyether Lupranol 3300 (OHV = 400 mg KOH·g^−1^) from BASF (Ludwigshafen, Germany) and propylene carbonate from Sigma-Aldrich (St. Louis, MA, USA) were used as liquefaction and oxypropylation agents, respectively.

The other reagents, including six functional commercial oxypropylated sorbitol based polyol-polyether for PUR foam processing Lupranol 3422 (OHV = 490 mgKOH∙g^−1^) from BASF (Ludwigshafen, Germany), commercial polymeric diphenylmethane diisocyanate (pMDI), with [NCO] = 7.5 mmol∙g^−1^ and an average functionality of 2.7 from BASF (Ludwigshafen, Germany), amine catalyst Polycat 5 (Evonik, Essen, Germany), the physical blowing agent Opteon™ 1100 (Chemours, Wilmington, DE, USA), and the surfactant Niax Silicone L-6915 (Momentive Performance Materials Inc., Leverkusen, Germany), were used for the creation of PUR foam compositions. The residual bark remaining after the isolation of extractives was introduced in PUR foam as a natural filler.

### 2.2. Characterization of BA Bark Biomass

2.2.1. Elemental analysis (C, H, N) of untreated, extracted bark, and extractives was performed using a vario MACRO elemental analyzer (ELEMENTAR Analysensysteme Gmbh, Langensebold, Germany), and the ash content was determined as a residue after ignition at 550 ± 5 °C in a Carbolite ELF 11/6 B furnace (Carbolite Gero, UK).

2.2.2 The content of different OH groups in extractives was examined using ^31^P NMR spectroscopy according to [46,47]. The average data of two repeated experiments were used for the discussion.

2.2.3 The non-isothermal thermal TG/DTG/DSC analysis in air and argon was performed in an alumina crucible on samples weighing approximately 20 mg using a Seteram Setline device (Seteram, Caluire-et-Cuire, France). The temperature range was set from 25 °C to 700 °C, with a heating rate of 5 °C per minute. Calisto 2.0 software was used for data processing. Three repeated experiments were performed for the composition under testing.

2.2.4 The FTIR spectra of extractives were generated using the KBr method. The Thermo Scientific Nicolet iS550 spectrometer (Norristown, PA, USA) was employed. The analysis was conducted in the range of 4000–400 cm^−1^ at a scan resolution and number of scans of 4 cm^−1^ and 32 scans, respectively.

### 2.3. Synthesis of Bio-Polyols

#### 2.3.1. The Liquefaction of BA Bark Extractives

A catalyst-free suspension of 50 g, consisting of Lupranol 3300 and extractives, was loaded into a 100 mL round-bottom flask equipped with a magnetic stirrer. The composition of each component was adjusted to vary the extractive content in the suspension within a range of 10–40%. The flask was placed in a silicone bath with automatic temperature control and stirred at temperatures of 125, 150 and 170 °C under a constant argon flow for 24 h The range of liquefaction temperatures was chosen based on the liquefaction of alder wood [48]. Three experiments were conducted under identical conditions. At intervals of 1, 3, 6, and 24 h, approximately 2.5 g of product was removed from the flask using a Pasteur pipette. Each aliquot was filtered through a glass filter (pore size 3) preheated to 120 °C. After filtration, the filter was cooled to room temperature, and the precipitate was washed with dichloromethane. The insoluble biomass was dried in an oven and then weighed. After 24 h, the remaining product in the flask was similarly filtered to separate the insoluble biomass from the bio-polyol. The bio-polyol portions obtained from the repeated experiments were combined and mixed. This sample was used for analysis and testing as a bio-polyol in PUR foam compositions. An enlarged batch of bio-polyol was prepared by liquefying 300 g of a 30% suspension at 150 °C for 6 h, followed by filtration of the insoluble fraction.

#### 2.3.2. Oxypropylation of BA Bark Extractives with PC

The “green” oxypropylation of BA bark extractives was performed under conditions previously developed by the authors and detailed in [33]. An enlarged portion of polyol was synthesized as follows: a mixture consisting of 100 g of extractives, 456 g of (PC), and 17 g of 8-diazabicyclo[5.4.0]undec-7-ene (DBU) as the catalyst, was loaded into a 1 L two-neck round-bottom flask equipped with a mechanical stirrer and a reflux condenser. The flask was placed in a silicone bath with temperature control and allowed to react at 150 °C with constant stirring and argon flow for 24 h. Completion of the reaction was confirmed by the disappearance of absorbance at ~1780 cm^−1^, attributed to the PC carbonyl group, in the FTIR spectra of the polyols. A total of 373 g of polyol was obtained, with a yield of approximately 65% based on the weight of the starting suspension.

### 2.4. Characterization of Bio-Polyols

2.4.1. The hydroxyl group content was determined by acetylating the samples with acetic anhydride and performing potentiometric titration of the liberated acetic acid with 0.1 N NaOH. Additionally, the content of phenolic and carboxylic groups in the bio-polyols obtained from the liquefaction of extractives was determined through conductometric titration of the bio-polyol’s alkali solution with 0.1 N HCl, using an automatic titration device (ABU 910), coupled with a Conductometer (CDM 210) and Titration Manager (TIM900) from Radiometer, Copenhagen, Denmark. The data from the potentiometric titration of the liquefied extractives were adjusted to account for the carboxylic group content [49]. The OHV of polyols was calculated according to [50].

2.4.2 The FTIR spectra analysis of bio-polyols was performed using the ATR technique. A Thermo Scientific Nicolet iS550 spectrometer (Norrison, PA, USA) equipped with ATR ZnSe and diamond crystals top plate was used. The analysis was performed in the range of 4000–400 cm^−1^ at a scan resolution and number of scans of −4 cm^−1^ and 32 scans, respectively.

2.4.3 The water content in bio-polyols was determined via Karl Fisher titration using the automatic titrator Model 275 KF (Denver Instrument, Bohemia, NY, USA).

2.4.4 The rheological measurements were made at 25 °C using the Anton Paar Modular Compact Rheometer MCR 92 (Anton Paar, Graz, Austria) with a cone-plate measuring system and a gap of 48 μm. Shear rate ramps were carried out from 1 to 100 s^−1^.

### 2.5. PUR Foam Preparation

PUR foams were obtained using the free-rising method with a two-component system consisting of a polyol mix and isocyanate.

The weight of pMDI was calculated using Equation (1):

M_pMDI_ = (M_1OH_/EW_1_ + M_2OH_/EW_2_ + 2M_H2O_/18) × 133.3 × 1.15 (1)

where

M_pMDI_—weight of pMDI (g);
M_1OH_—weight of polyol 1(g);
M_2OH_—weight of polyol 2 (g);
M_H2O_—weight of water in system (g);
18—molar weight of water (g∙mol^−1^);
EW_1_—equivalent weight of polyol 1 (g∙Eq^−1^);
EW_1_—equivalent weight of polyol 2 (g∙Eq^−1^);
133.3—equivalent weight of pMDI (g∙Eq^−1^);
1.15—molar NCO/OH ratio.

To prepare the polyol system, 30 g of any or mix of two polyols was loaded into a paperboard cup shaped like an inverted cone (d_1_ = 72 mm; d_2_ = 97 mm, H = 180 mm). A specified amount of amine catalyst (Polycat 5), surfactant (Niax Silicone), and water was added, followed by thorough mixing at 2000 rpm for approximately 1 min using a high-speed mechanical stirrer. The blowing agent, Opteon™ 1100, was then added as the final ingredient, and the polyol system was premixed again at 2000 rpm for 15–20 s. Afterward, pMDI was added in an amount sufficient to achieve an NCO/OH molar ratio of 1.15, and the mixture was stirred for 10 s. Foaming was carried out in the same cup.

The foam rise dynamic was monitored using a Qualification System FOAMAT 285 [51].

The same compositions were used to prepare PUR foams filled with extracted bark. In this case, the required amount of filler was added to the polyol system (without the blowing agent) and premixed for about 30 s using a high-speed mechanical stirrer. The blowing agent and pMDI were then added, followed by mixing all ingredients as previously described. After 24 h of post-curing at room temperature, the excess foam above the cup was trimmed off. In all cases, samples for PUR foam testing were taken from a 65 mm-deep layer of material located below the top of the cup.

### 2.6. Characterization of PUR Foams

2.6.1 The apparent density was measured according to ISO 845:2006.

2.6.2 The closed-cell content by volume was measured and calculated according to ISO 4590:2016. The helium pycnometer AccuPyc II 1340 (Micrometrics, Norcross, GA, USA) was used. Three samples were tested for each PUR foam composition.2.6.3 The thermal conductivity coefficient (λ) was measured using a FOX 200 heat flow meter (TA Instruments, New Castle, DE, USA) at an average temperature of 10 °C (cold plate: 0 °C, and hot plate: +20 °C, sample dimensions: 200 mm × 200 mm × 40 mm), following the ISO 8301:1991 standard.

2.6.4 A scanning electron microscope SEM TESCAN TS 5136 MM (Tescan Vega, Ottawa, ON, Canada) was used to study the cell structure of PUR foams.

2.6.5 The compressive strength and modulus of PUR foams in the foaming direction were determined according to ISO 844:2021. A Zwick/Roell Z100 universal testing machine (Zwick Roell, Ulm, Germany) was used. The maximum test load was 1 kN, with a deformation rate of 10% of the sample height per minute. Six cubic samples, 30 mm in size, were tested for each PUR foam composition.

2.6.6 An FTIR spectra analysis of PUR foam samples, previously ground in a cryogenic mill Retsch CryoMill (Retsch, Haan, Germany), was performed using the KBr method, following the procedure described in Section 2.2.4.

2.6.7 For non-isothermal TG/DTG of PUR foam in air, ground PUR foam samples, prepared using a cryogenic mill, were tested. A non-isothermal thermal analysis in air was performed similarly to the procedure described in Section 2.2.3.

### 2.7. Pretreatment of Extracted BA Bark for Use as a Filler in the PUR Foam Compositions

The extracted bark was air-dried to an approximately 10% moisture content and then ground in a Fritsch Pulverisette 5/2 planetary mill (Berlin, Germany) for 1 h, followed by oven drying at 100 °C for 24 h before being incorporated into the PUR foam compositions.

### 2.8. Filler Characterization

2.8.1 Sieve analysis of the powder was performed using an electromagnetic sieve shaker (CISA Cedaceria Industrial, Barcelona, Spain) with a set of mesh sieves (cell sizes: 200 µm, 100 µm, and 50 µm, respectively).

2.8.2 A scanning electron microscope (SEM) Tescan TS 5136 MM was used to evaluate the size and shape of the filler particles.

2.8.3 The BET surface area and total pore volume of the powder were measured using N_2_ sorption with a Quantachrome Instrument (Anton Paar QuantaTec, Boynton Beach, FL, USA).

## 3. Results and Discussion

### 3.1. The Characteristics of BA Bark Biomass

The extractives, totaling approximately 250 g, were obtained through the short-time microwave-assisted water extraction of black alder (BA) bark at 90 °C, as described in [44]. Ten repeated experiments were conducted, yielding an average extractive content of 15.8 ± 1.0% on a dry matter (DM) basis. The proximate analyses of the starting bark, extractives, and residual bark are presented in Table 1.

A significant decrease in the ash content of the extractives compared to the parent bark was observed, which can be considered a beneficial factor when considering the valorization of BA extracts as precursors for polyol synthesis. The fixed carbon in extractives was one-half-fold higher than in untreated bark. This indicates that the isolated extractives are more enriched with aromatic compounds compared to the parent bark (Figure S1 in the Supplementary Materials, Table 1).

Earlier, authors reported, using HPLC analysis, that the water extractives of BA bark consist of various non-lignin polyphenolics, including diarylheptanoids, condensed tannins, flavonoids, and carbohydrates and organic acids [46] (Table S1 in the Supplementary Materials). Oregonin, which is the xyloside form of the diarylheptanoid, was the dominant component of the extractives, with a concentration of approximately 31% (Figure S2 in the Supplementary Materials). The total content of monomeric carbohydrates in the extractives after complete hydrolysis determined via GC was about 38%. Among them, 25% was a xylose derived in the results of oregonin hydrolysis. The average molecular weight number (*Mn*) of the extractives, ~1100 Da, indicated that the carbohydrates and condensed tannins present in the extractives are predominantly in oligomeric form [33,52,53].

**Figure 1:**
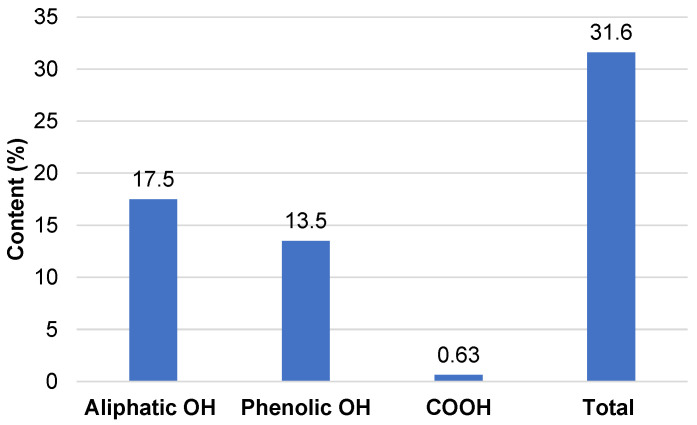
The content of OH groups of different originations in BA bark extractives according to ^31^P NMR.

The concentration of OH groups is a key characteristic of any precursor suitable for polyol processing. The OH group content in the BA extract was evaluated using ^31^P NMR (Figure 1).

Taking into account the ^31^P NMR data, the OHV of extractives were 1042.8 mgKOH∙g^−1^, 57% of which was presented by phenolic groups. It was proposed that, compared to the parent bark, the absence of high-molecular-weight phenolic and carbohydrate components typical of lignocellulosic cell walls, along with the low equivalent weight and high accessibility of OH groups in extractives, will favor the processing of extractives into liquid bio-polyols.

### 3.2. Liquefaction of BA Bark Extractives

#### 3.2.1. The Yield of Liquefaction and Dependence on Processing Conditions

The liquefaction of extractives was performed using tri-functional polyether polyol Lupranol 3300 obtained in the industry via the oxypropylation of glycerol with PO and used in general rigid PUR foam compositions.

The averaged molecular weight number (*Mn*) of Lupranol 3300 of 421 g∙mol^−1^ was calculated using Equation (2) [51]:

(2) $$M_{n}=\frac{f\times 56,100}{OHV}$$

where

*Mn*—number average molecular weight, g∙mol^−1^;
*f*—the number of OH groups per mol, equal 3;
56,100—equivalent weight of KOH, mg∙Eq^−1^;
*OHV*—hydroxyl value of Lupranol 3300, equal to 400 mgKOH·g^−1^.

**Figure 2:**
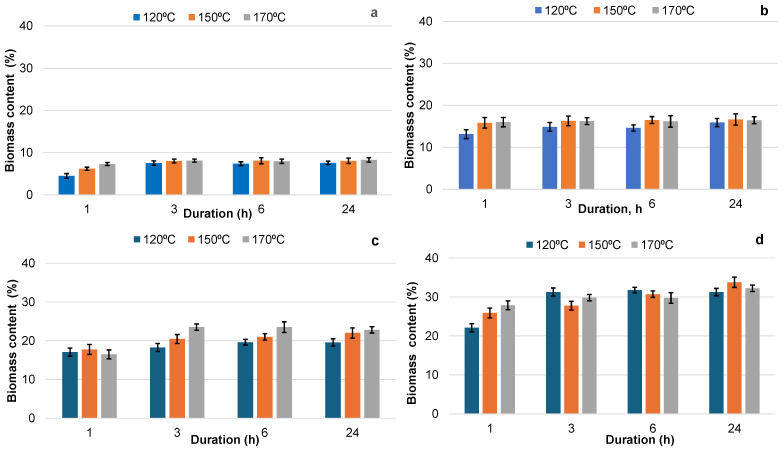
The content of liquefied biomass in bio-polyols as a function of the duration and temperature of processing at different biomass contents in the starting suspension: 10% (**a**), 20% (**b**), 30% (**c**), 40% (**d**).

Therefore, in the bio-polyols obtained, we can characterize BA bark extractives as higher molecular weight (*Mn* = 1100 g∙mol^−1^) and highly functional components, more enriched with OH groups compared to Lupranol 3300, which served as the low-molecular hydroxyl-containing solvent with a molecular weight similar to PEG 400, the most widely used solvent for biomass liquefication [39]. Since the main components of the extractives, including phenolics and carbohydrates, are present as monomers and oligomers, sulfuric acid was not used as a catalyst. This allows the process of extractive liquefaction to be characterized as more environmentally friendly vs. the liquefaction of bark and other lignocellulosic biomass that is catalyzed by acids or alkali [54]. It was shown that as the biomass content in the suspension increased from 10% to 40%, the content of liquefied biomass in the final bio-polyols steadily rose from 8% to 32% (Figure 2).

**Figure 3:**
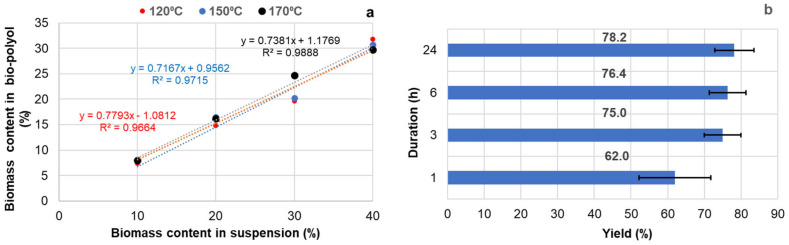
Plots of liquefied biomass content in bio-polyol vs. biomass content in the initial suspension after 6 h of extractive liquefaction at different temperatures (**a**), and the effect of the duration on the yield of liquefied BA bark extractives, independent of the temperature and biomass content in the initial suspension (**b**).

The yield of liquefaction was defined as the weight ratio of biomass soluble in Lupranol 3300 at any given point during processing to the weight of extractives in the initial suspension. It was established that the liquefaction yield was less dependent on the suspension concentration and processing temperature when the duration was 3, 6, or 24 h. In this case, a similar slope was observed in the plots showing the correlation between the biomass content in the final bio-polyols and biomass content in the initial suspension, regardless of the liquefaction temperature (Figure 3a).

For each of the mentioned durations, a similar liquefaction yield in the range of 75–78% was achieved, independent of the temperature and biomass content in the initial suspension. The coefficient of variation (CV) for the average liquefaction yield under different conditions was about 7% (Figure 3b). Under the same conditions, the average liquefaction yield after 1 h of processing was notably lower, at 62%, with a comparatively higher CV of 15.2%, indicating that the process had not yet stabilized (Figure 3b). Based on these results, we determined that a duration of 6 h and a temperature range of 120–150 °C were optimal for the liquefaction of BA bark extractives in Lupranol 3300 in terms of the liquefaction yield.

#### 3.2.2. The Characteristics of Bio-Polyols Generated via Liquefaction of BA Bark Extractives in Lupranol 3300

**Figure 4:**
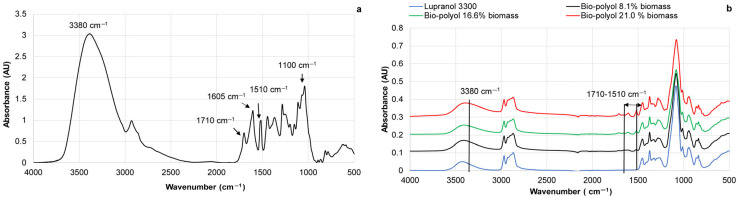
FTIR spectra of BA extract (**a**) pure Lupranol 3300 and bio-polyols with varying biomass contents liquefied at 150 °C for 6 h (**b**).

The FTIR spectra of BA bark extractives, pure Lupranol 3300, and bio-polyols with different contents of biomass are presented below (Figure 4a,b).

**Figure 5:**
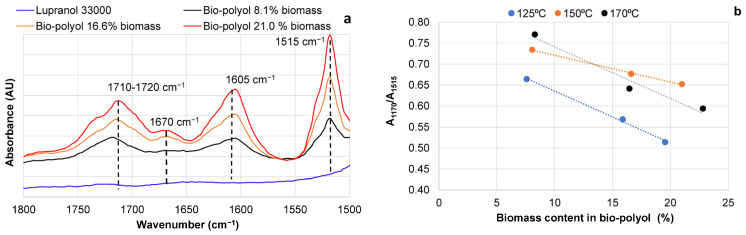
FTIR spectra in the region of 1800–1500 cm^−1^ of pure Lupranol 3300 and bio-polyols with varying biomass contents liquefied at 150 °C during 6 h (**a**) and FTIR spectra absorbance ratio (A_1710 cm_^−1^/A_1515 cm_^−1^) of bio-polyols synthesized at different temperatures vs. the biomass content in them (**b**).

In contrast to the spectra of pure Lupranol 3300, the FTIR spectra of bio-polyols obtained showed absorbances in the region of 1710–1510 cm^−1^, with the peak at 1710–1720 cm^−1^ attributed to unconjugated carbonyl (Figure 4a and Figure 5a). The others peaks at ~1605 cm^−1^ and ~1515 cm^−1^ are typical of aromatic skeletal vibrations (Figure 4a and Figure 5a). These absorbances appeared, indicating the successful solubility of the biomass portion in Lupranol 3300 under the conditions studied. An increase in the intensity peaks at 3380 cm^−1^ was observed with an increasing soluble biomass content (Figure 4b). Due to the higher OHV of the extractives (1042.8 mg KOH∙g^−1^) compared to Lupranol 3300 (400 mg KOH∙g^−1^), the steady growth in the intensity of these peaks with an increasing soluble biomass content suggests that the bio-polyols are more enriched with hydroxyl groups in comparison to Lupranol 3300.

Due to the high chemical and thermal stability of aromatic rings, the variation in the absorbance intensity at 1515 cm^−1^ in bio-polyols can be attributed to changes in the aromatic content resulting from the liquefaction of phenolic-rich extractives. In contrast, the increase in absorbance intensity at 1710 cm^−1^ in the bio-polyols may be attributed not only to the presence of carboxyl groups in the non-liquefied extractives but also to the partial oxidation of carbohydrate moieties present in the extractives during processing [55,56].

For example, the thermal dehydration of glucose is accompanied by the formation of hydroxymethyl furfural, which is then followed by the production of levulinic acid [56]. The ratio of the absorbance intensity at ~1710 cm^−1^ to that at 1515 cm^−1^ (aromatic skeletal vibration), used as an internal standard in the FTIR spectra of bio-polyols, indicates that the extent of oxidation of the biomass components depends on the liquefaction conditions (Figure 5b). The greatest oxidation was observed at the lowest concentration of extractives in bio-polyols, i.e., the highest Lupranol 3300/extractives ratio. The A_1710_/A_1515_ ratio decreased with an increasing biomass content in the bio-polyol. Oxidation was more pronounced at 150 °C and 170 °C compared to 120 °C. The small absorbance at 1670 cm^−1^ in the FTIR spectra of bio-polyols indicates that the formation of quinone moieties as a result of the oxidation of hydroxyl groups in catechol units from oregonin, other diarylheptanoids, and tannins takes place [57]. In this case, the nucleophilic addition of hydroxyl groups from Lupranol 3300 to quinone groups, leading to the formation of dialkoxy benzoquinone, could indeed be a plausible mechanism of the chemical interactions between the extractives and solvent [58]. This modification can influence the solubility and other chemical properties of extractives within the solvent matrix, resulting in the increasing viscosity of polyols. On the other hand, condensation products can participate in the formation of PUR matrices as a single macromonomer, positively influencing their mechanical and thermal properties.

The results obtained were confirmed through the functional analysis of synthesized bio-polyols. The content of phenolic and carboxylic groups in the bio-polyols was determined separately using acid–base conductometric titration [49]. The experimental data were compared with calculated values based on the additivity principle, taking into account the functional analysis data of extractives using ^31^P NMR, assuming no functional transformation of the biomass.

**Figure 6:**
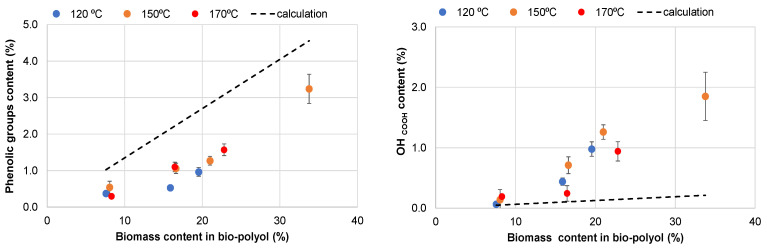
Experimental and calculated content of phenolic and OH_COOH_ groups (in bio-polyols obtained at different temperatures) dependent on the liquified biomass content.

It was shown that the experimentally determined OH_COOH_ contents exceeded the calculated values (Figure 6). Conversely, the experimentally determined phenolic group content in the bio-polyols was lower than the calculated values. These findings align with the FTIR results, suggesting the partial oxidation of catechol units and the possible transformation of carbohydrates into organic acids.

The primary characteristic of bio-polyols used in PUR production is their hydroxyl group content, typically expressed as OHV. Another important feature related to bio-polyol functionality is the content of carboxylic groups, expressed as the acid number [50]. The acid number is crucial for adjusting the OHV to accurately determine the actual hydroxyl group content in the bio-polyol, typically corrected by adding OHV values measured using acetylation methods. Additionally, carboxylic groups reduce the activity of tertiary amine catalysts through acid–base neutralization. In PUR foam compositions, carboxylic acids also act as chemical blowing agents, reacting with isocyanates to form amides and carbon dioxide [59]. Consequently, an increase in the extractive content of bio-polyols may lead to decreased activity in PUR foam systems, along with a slightly lower foam density.

**Figure 7:**
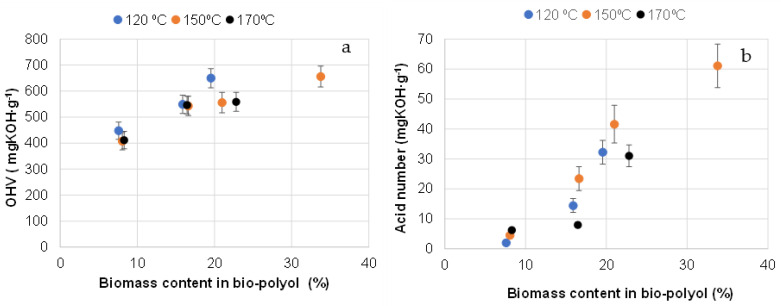
OHV (**a**) and acid numbers (**b**) of bio-polyols obtained at different temperatures and dependence on the liquified biomass content in them.

As demonstrated, the OHV of the bio-polyols obtained ranged from 410 to 650 mg KOH∙g^−1^, compared to 400 mg KOH∙g^−1^ for pure Lupranol 3300. The OHV and acid number increased with a higher biomass content in the bio-polyol (Figure 7a,b). At the same biomass content, bio-polyols synthesized at higher temperatures showed slightly lower hydroxyl group contents than those synthesized at 120 °C. This is likely due to the more significant condensation reactions between the hydroxyl groups of the polyether and biomass components at elevated temperatures [2] (Figure 7a).

**Figure 8:**
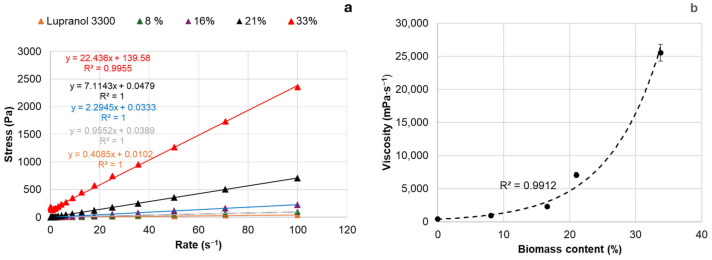
Flow curves (**a**) and dynamic viscosity at 25 °C (**b**) of bio-polyols with different contents of biomass synthesized at 150 °C.

Viscosity is one of the key parameters of bio-polyols used in PUR synthesis, as high-molecular-weight PUR is obtained using low- or medium-viscosity liquid intermediates [50]. The low viscosity of bio-polyols offers a technological advantage, broadening their practical applications. Generally, the OHV of commercial polyol polyethers used for PUR rigid foam production ranges from 300 to 800 mg KOH∙g^−1^, with dynamic viscosity reaching up to 30,000 mPa∙s at 25 °C [2]. As shown, as the biomass content increases up to 30–33%, the viscosity of the bio-polyols increases exponentially from approximately 950 to 25,550 mPa∙s (Figure 8b). This suggests that condensation between extractives and hydroxyl groups in the bio-polyols occurs, an effect that becomes more pronounced at higher biomass contents. At a biomass content of 21%, the viscosity was significantly lower, around 7000 mPa∙s. The bio-polyols produced by liquefying extractives with Lupranol 3300 in ratios ranging from 1:9 to 3:7, followed by the removal of insoluble biomass, are uniform, viscous liquids free from biomass aggregates. As a result, the viscous stresses during bio-polyol flow are linearly correlated with the deformation rate over time, allowing them to be classified as Newtonian fluids (Figure 8a).

As the ratio of extractives to Lupranol 3300 increased to 4:6, a deviation from Newtonian behavior was observed, which was accompanied by a drastic increase in polyol viscosity (Figure 8b).

Based on this suggestions, bio-polyol with a biomass content of 21.0 ± 0.5%, synthesized by liquefying extractives with Lupranol 3300 at a weight ratio of 3:7 at 150 °C for 6 h, was tested in compositions for rigid PUR foams. An enlarged batch of this bio-polyol was prepared by liquefying 300 g of extractives, yielding the following characteristics: OHV = 539 mg KOH∙g^−1^, acid number = 35 mg KOH∙g^−1^, viscosity at 25 °C 7350 mPa∙s at 25 °C, and water content of 0.25%.

### 3.3. The “Green” Oxypropylation of BA Bark Extractives with PC and Characteristics of Ensuing Polyols

The study of the oxypropylation of BA bark extractives using PC as a “green” alternative to fossil-derived PO in the presence of DBU was previously described by the authors in [33]. It was demonstrated that under optimal conditions—a PC/OH molar ratio of 3, DBU/OH molar ratio of 0.1–0.075, and a reaction duration of 24 h at 150 °C—uniform polyols with viscosities of around 1000 mPa·s^−1^ were synthesized. Under these conditions, the extractive components react with an equivalent amount of PC to form copolymers, while excess PC produces oligopropylene diols, which lower the polyol viscosity and act as bifunctional co-reagents in PUR foam systems. Notably, the high content of phenolic groups in the extractives promoted copolymerization. Both processes predominantly proceed via an etherification mechanism, with the release of carbon dioxide (Figure S3 in the Supplementary Materials). Reducing the PC/OH ratio below 3 was not feasible due to the incomplete wetting of the extract by the PC, which serves as both the solvent and reagent. Increasing the proportion of PC decreases the biomass content in the polyols and extends the reaction time required for the complete conversion of the reagents. In this study, a larger batch of polyol was synthesized by repeating the previously established optimal conditions (Table 2).

It should be noted that the tertiary amine DBU (Figure S4 in the Supplementary Materials), present in the polyol and initially used as a catalyst for the anionic polymerization in the reaction of PC with extractives, can be reused as a catalyst for urethane formation in PUR foam systems [52].

### 3.4. Characteristic of Extracted BA Bark and Its Pretreatment for Utilization as a Natural Filler in Rigid PUR Foam Compositions

#### Proximate and TG/DTG Analysis

About 85% of BA bark in dry matter (DM) remained as a solid residue after microwave-assisted extraction with water. In this study, the residual bark was characterized in terms of its elemental composition, ash content, thermal characteristics under oxidative and inert atmospheres, particle size, and surface properties, with a focus on applying the residual biomass as a filler for rigid PUR foam compositions developed from BA bark extractive-derived polyols. This approach aligns with the biorefinery concept, which aims for the complete and most profitable utilization of biomass within the technological cycle.

**Figure 9:**
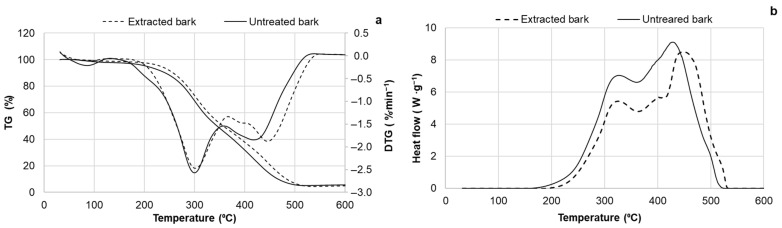
The DTG (**a**) and DSC (**b**) curves of untreated and extracted bark in air media.

As was shown above, the proximate analysis revealed a similar elemental composition between the extracted bark and the initial biomass, with a slight decrease in the ash content (Table 1). Additionally, the untreated and extracted bark exhibited similar behavior at high temperatures in an inert atmosphere. As was shown above (Table 1) for argon, the thermal degradation of the bark occurred at a maximum rate of around 350 °C, leaving a residual fixed carbon content of approximately 27% (Figure S1). However, TG/DSC analysis in air showed some differences in the degradation patterns of the untreated and extracted bark regarding the mass–energy balance (Figure 9a,b).

For both samples, the amount of heat released during volatile oxidation (200–350 °C) was lower than that released during char combustion (350–520 °C) (Figure 9b). In contrast, the major portion of biomass was degraded in the 200–350 °C range. It was calculated that the yield of volatiles, 53.5% based on the DM of extracted bark, was accompanied by the release of approximately 33.5% of the total heat, with an energy/mass ratio of 0.62. For untreated bark, this coefficient was 0.73, as the yield of volatiles, 56.2%, was accompanied by the release of 41% of the total heat. This indicates a comparatively lower calorific value for the volatiles produced during the thermal oxidative degradation of extracted BA bark compared to untreated bark. Therefore, regarding the influence on the flame-retardant properties of PUR foams, we propose that incorporating extracted bark residues as a filler could be more suitable than using untreated bark. This is primarily due to the release of lower-calorific-value volatiles under high-temperature conditions (200–350 °C).

**Figure 10:**
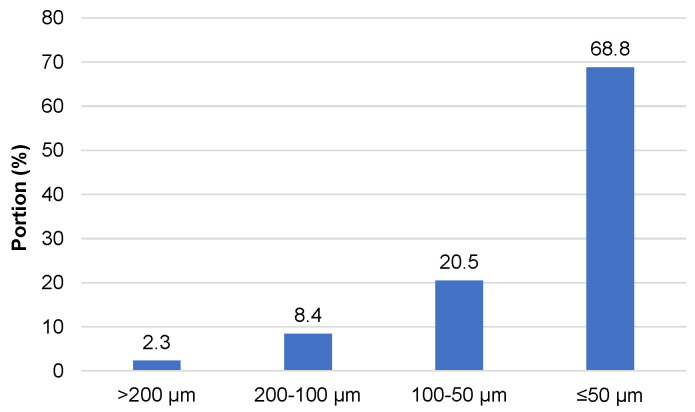
The particle size distribution of ground-extracted bark.

To introduce the extracted bark into the PUR foam composition as a filler, it was air dried up to ~10% of the water content, followed by grinding in a planetary mill. The sieve analysis of grind powder was performed using an electromagnetic sieve shaker and complect of mesh sieves with cell sizes of 200 µm, 100 µm, and 50 µm, respectively (Figure 10).

**Figure 11:**
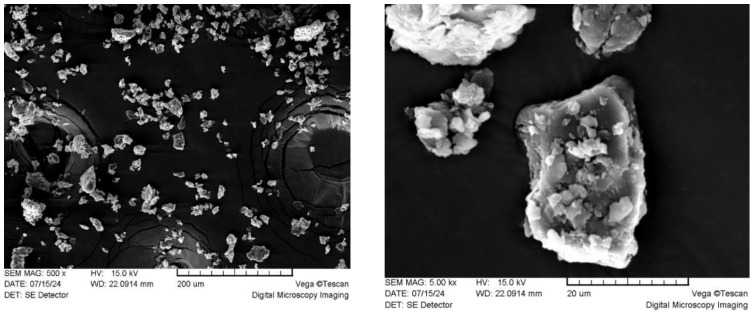
SEM image of ground BA bark at different magnifications: ×500 (**left**) and ×5000 (**right**).

As was shown, 98% of particles were below 200 µm. The dominant fraction of size was equal to or below 50 µm with an irregular shape (Figure 10 and Figure 11).

The measured total pore volume of the powdered extracted bark was relatively low, at approximately 3.7 mm^3^∙g^−1^, indicating an underdeveloped pore system in the filler particles. Consequently, the specific BET surface area measured at 1.03 m^2^∙g^−1^ is primarily attributed to the outer surface of the particles, which may be available for adhesion interactions with the PUR matrix through the formation of physical bonds.

### 3.5. Rigid PUR Foams Based on BA Bark Extractive-Derived Polyols

#### 3.5.1. Compositions of PUR Foams

In this study, two PUR foam compositions based on Lupranol 3300 and a mix of Lupranol 3300 and Lupranol 3422 with abbreviations of Ref. 1 and Ref. 2, respectively, were used as a reference (Table 3). Five rigid PUR foam compositions containing bio-polyols were prepared for testing (Figure S5 in the Supplementary Materials). The partial or complete substitution of commercial polyols with synthesized bio-polyols (BP) was performed. In compositions abbreviated as BP-1 and BP-2, three functional polyol Lupranol 3300s were substituted with oxypropylated and liquefied extractives, respectively, with the remaining 30 parts by weight (pbw) of high functional polyol Lupranol 3422 in composition. In recipes BP-3 and BP-4, both commercial polyols were substituted with oxypropylated and liquified extractives. Finally, in the BP-5 formulation, both commercial polyols were substituted with a mix of both bio-polyols in equal proportions (Table 3).

In compositions containing oxypropylated extractives, catalyst Polycat 5 was not added due to the presence of the DBU catalyst remaining in the bio-polyol after oxypropylation.

The effect of extracted bark as a filler on the foaming process and properties of the material were studied using three PUR foam compositions: one based on a commercial polyol mix, one based on oxypropylated extractives, and one based on a mix of an oxypropylated and liquefied biomass. The filled compositions are abbreviated as Ref. 2/F, BP-3/F, and BP-5/F (Table 4).

In our study, we assumed that the extracted bark would act as a non-reactive filler. Therefore, no adjustments were made to the pMDI consumption based on the OH group content in the extracted bark.

The filler was introduced into the formulation at a ratio of 10–40 pbw per 100 pbw of polyol, meaning that its content in the final material ranged from 3.6% to 13.9%. Increasing the filler content above 40 pbw resulted in a high viscosity of the polyol system, which hindered the proper mixing of ingredients.

As was shown, the content of bark biomass in the PUR matrix varied in the range of 5.8–11.1% depending on the extent of commercial polyol substitution and the type of bio-polyols used (Table 3). By using extracted bark as a filler in combination with bio-polyol, the portion of bark biomass recalculated based on the PUR matrix was increased up to 22–24% (Table 4, Figure S6 in the Supplementary Materials).

#### 3.5.2. Dynamics of PUR Foam Rising

**Figure 12:**
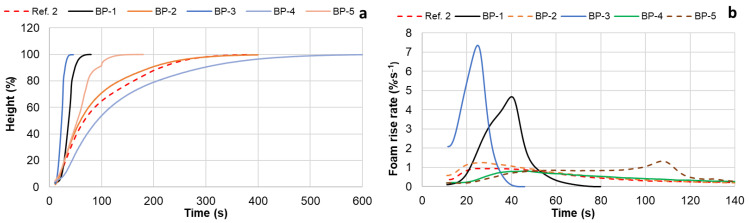
The height (**a**) and rise rate (**b**) of PUR foams versus time.

The rise in PUR foams was studied using the Universal Foam Qualification System FOAMAT 285. For an easier comparison, for all compositions, the foam height was expressed as a percentage of its maximum value (Figure 12a,b).

The activity of both reference compositions was similar; therefore, only the height rise dynamics of the Ref. 2 sample are presented in the graph. As shown, the BP-3 composition, with the complete substitution of both commercial polyols with oxypropylated extractives, exhibited the highest activity. It had a foam rise time of approximately 45 s and a maximum foam rise rate of 7.2% per minute, reaching 25 s after blending the ingredients. This foaming dynamic appears to be more suitable for spray systems. As mentioned earlier, this can be attributed to the comparatively high DBU content in the polyol (Table 2) and the grafting of oxypropyl units onto extractive components, which eliminates the electronic and steric constraints of the OH groups in the resulting copolymer during the urethane formation reaction [29].

In contrast, another PUR foam composition, BP-4, containing only liquefied extractives as the bio-polyol, was the least active, with a maximum foam rise rate of 0.9% per minute and a foam rise time of about 550 s. This can primarily be explained by the lower concentration of the amine catalyst in this composition compared to the others due to its higher B/A ratio (Table 3). For instance, to achieve a constant NCO/OH ratio of 1.15, the B/A ratios for the Ref. 1, Ref. 2, and BP-4 compositions are 0.97, 1.03, and 1.28, respectively (Table 3). This means that the BP-4 formulation has about a 20–25% lower weight concentration of the amine catalytic system Polycat 5 compared to the references.

Additionally, the presence of acidic carboxylic and phenolic groups, which are less reactive with isocyanates and can neutralize amine catalysts in liquefied extractives, may also contribute to the lower reactivity of liquefied extractives compared to other polyols. Mixing both bio-polyols in equal proportions significantly decreases the foam rise rate in the BP-5 composition, resulting in a foam rise time of about 150 s compared to the BP-3 formulation, but significantly increases it compared to the BP-4 formulation (Figure 12a,b).

Interestingly, unlike other PUR foam systems, the differential curve of the BP-5 foam rise shows two peaks (Figure 12b). This may be explained by the high ability of DBU, a base, to form complexes with the acidic groups present in the liquefied biomass. In this case, the first, lower-rate peak is attributed to the reaction between the isocyanate and the DBU–acidic OH group complex, which forms first but is less reactive due to the low nucleophilicity of the carboxylic and phenolic OH groups. As the catalyst is liberated, the formation of the DBU–aliphatic OH group complex occurs, leading to a higher-rate reaction with NCO, which is responsible for the second peak. Another possible explanation for the second peak could be the partial homopolymerization of isocyanate, forming carbodiimide and/or isocyanurate units under these conditions. Further investigation is needed to clarify this effect.

The results presented above indicate that by varying the proportion of oxypropylated and liquefied extractives in polyol mixtures, the dynamics of rigid PUR foam rise can be controlled over a wide range without the addition of catalysts.

Introducing commercial Lupranol 3422 into the composition, based on oxypropylated and liquefied extractives, resulted in retardation of the foam rise process in the first case (BP-1) and an acceleration in the second (BP-2) (Figure 12a,b).

**Figure 13:**
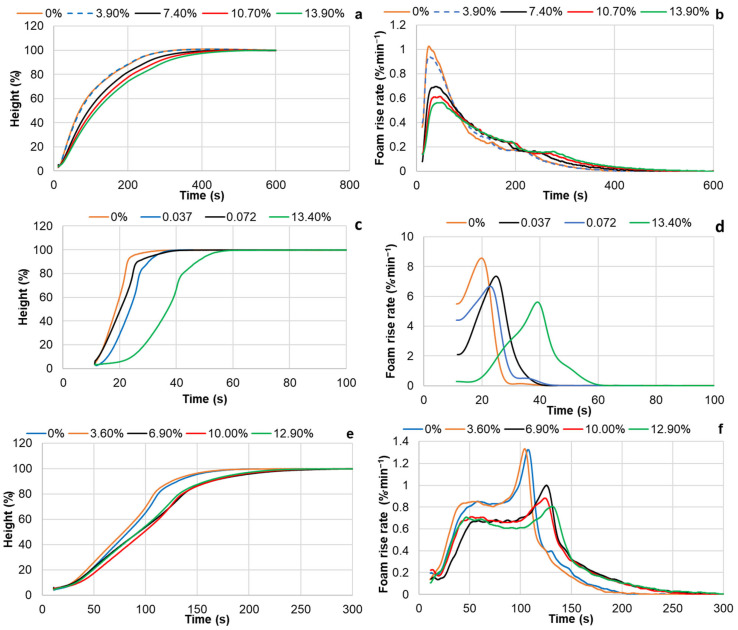
Effect of extracted bark content as a filler in PUR foam on the height (**a**,**c**,**e**) and foam rise rate (**b**,**d**,**f**) across different PUR foam compositions: Ref. 2 (**a**,**b**); BP-3/F (**c**,**d**); and BP-5/F (**e**,**f**).

A similar retardation effect of the filler, with an increasing content in the formulation, on the foam rise dynamics was observed in both reference and bio-polyol-based PUR foams (Figure 13a–f). This confirms the assumption made above about the role of this filler as an inert ingredient for which the introduction reduces the concentration of reactive components in the PUR foam compositions.

#### 3.5.3. Structural Characterization of PUR Foams via FTIR

**Figure 14:**
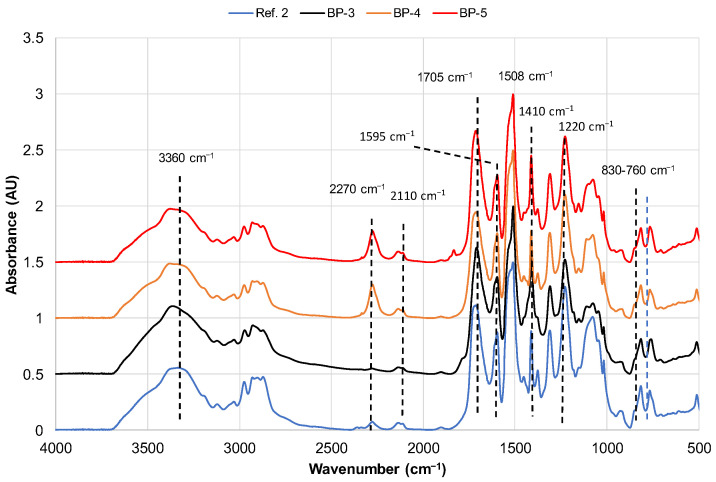
FTIR spectra of rigid PUR foams.

The FTIR spectra of reference PUR foam based on a Lupranol 3300 and Lupranol 3422 mix (Ref. 2), along with PUR foams based on bio-polyols, including oxypropylated extractives (BP-3), liquefied extractives (BP-4), and their combination (BP-5), are presented below (Figure 14).

The absorbance bands typical for PUR structures, obtained through the condensation of polyol with aromatic diisocyanates, were observed in the spectra of both reference and bio-polyol-based PUR foams [21,60]. The FTIR spectra showed strong absorbance at approximately 3360 cm^−1^ (amide A band) and 1508 cm^−1^ (amide II band), attributed to N-H vibration in urethane bonds; a strong and broad absorbance at ~1705 cm^−1^ (amide I band) due to the stretching vibration of hydrogen-bonded urethane carbonyl (C=O); at 1220 cm^−1^ attributed to C–O stretch in urethane; and at ~1595 cm^−1^ and 830–760 cm^−1^, respectively, due to skeletal ring (C–C) breathing mode and C–H out of plane vibration in aromatic rings.

In all PUR foam compositions, a small excess of pMDI was used (Table 3). Consequently, some peaks attributed to the self-condensation products of isocyanate units were observed. For instance, the absorbance at 1410 cm^−1^ is generally attributed to C–N stretching in isocyanurate or uretidinedione rings [61,62]. Additionally, an absorbance at ~2110 cm^−1^, attributed to carbodiimide (R_1_–N=C–R_2_), was present in all PUR foam compositions [62].

The presence of unreacted NCO groups in PUR foam is a commonly observed phenomenon [62,63] often explained by diffusion restrictions that occur as the cross-linking density in the polymeric matrix increases [64]. The band corresponding to the unreacted NCO group at ~2270 cm^−1^ was practically absent in the PUR foam based on oxypropylated extractives, yet present in the FTIR spectra of other foam compositions, including the reference sample. This may be due to the high reactivity of the bio-polyol system, which is based on oxypropylated extractives with isocyanate. This reactivity is likely due to the residual ~4% DBU remaining in the polyol after the modification of extractives with PC (Figure 12a,b; Table 2) and the high availability of OH groups in the biomass due to PO grafting, as previously mentioned [33].

The peak of unreacted isocyanate was more pronounced in compositions containing liquefied extractives, likely due to the lower reactivity of OH groups in liquefied extractives with isocyanate compared to oxypropylated extractives. Moreover, we propose that the average functionality of these polyols, which contain a trifunctional co-reagent, is higher compared to that of oxypropylated extractives containing bifunctional propylene glycols as a co-reagent. Consequently, diffusion restrictions due to gel formation emerge at a lower NCO conversion, resulting in a more significant portion of unreacted isocyanate.

#### 3.5.4. Apparent Density and Compression Properties of Rigid PUR Foams

**Figure 15:**
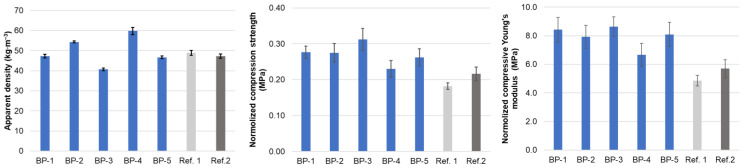
Apparent density, normalized strength, and Young’s modulus under axial compression parallel to the foaming direction for reference and bio-polyol-based rigid PUR foams (sample abbreviations are consistent with Table 3).

The apparent density is a crucial characteristic of rigid PUR foam that strongly influences the physical and mechanical properties of the material [65,66]. In this study, a combination of the chemical blowing agent water and the physical blowing agent Opteon™ was introduced into the polyol system, focusing on synthesizing PUR foams with an apparent density of 50 ± 10 kg∙m^−3^, suitable for applications in lightweight construction and thermal insulation materials (Figure 15).

The lowest density of approximately 40 kg∙m^−3^ was observed for the composition BP-3, based on oxypropylated extractives (Figure 15). The highest apparent density of around 60 kg∙m^−3^ was found for the BP-4 PUR foam, which contains liquefied extractives as the sole polyol (Table 3). BP-3 exhibited the highest activity (Figure 12a,b), evident through its elevated rate of heat release during foaming, which must be accompanied by the highest temperature within the foaming block and an increased volume of freon vapor. This resulted in a greater volumetric proportion of the gas in the cured material compared to other PUR foam systems [33,63]. In contrast, the BP-4 composition demonstrated the lowest activity, with a reduced rate of heat release and the highest volumetric proportion of PUR in the cured material. Additionally, the high B/A ratio of 1.28 suggests a lower weight concentration of blowing agents in this formulation compared to others, which contributes to the increased apparent density of the PUR foam (Table 3). This occurs despite the elimination of CO_2_ due to interactions between the carboxylic groups and isocyanate.

The apparent density of other bio-polyol-based PUR foams ranged from 46 to 54 kg∙m^−3^, closely matching that of the reference PUR foams, which varied from 47 to 49 kg∙m^−3^.

To accurately assess the effect of bio-polyols on the compressive properties of the cured material, the effect of density was normalized. The compression strength (σ) and Young’s modulus (E) were normalized to a density of 40 kg∙m^−3^ using Equations (3) and (4) [67].

(3) $$\sigma_{norm}={\sigma_{exp}(40/\rho_{exp})}^{2.1}$$

(4) $${E_{norm}=E_{exp}(40/\rho_{exp})}^{1.7}$$

where

*σ_norm_*, *E_norm_*—normalized compression strength and Young’s modulus, respectively (MPa);
*σ_exp_*, *E_exp_*—experimental compression strength and Young’s modulus, respectively (MPa);
ρ*_exp_*—experimental apparent density (kg m^−3^).

It was shown that samples of all PUR foam compositions containing bio-polyols exhibited significantly higher compression characteristics than those of the reference composition (Figure 15). The reference formulation, Ref. 1, based solely on the trifunctional polyol Lupranol 3330, had the lowest values for both strength and modulus. This suggests that the increased average functionality of polyols synthesized through oxypropylation and liquefaction methods using BA bark extractives—with high OH groups content and comparatively low molecular weights—resulted in greater crosslinking within the PUR matrix. Obviously, this higher crosslinking is a key factor contributing to the enhanced compression properties of bio-polyol-based PUR foams.

The BP-3 composition demonstrated the best characteristics, particularly in terms of the compression strength (0.31 MPa), which meets the CS(10/Y)300 level for compressive strength according to EN-14315-1 standards. These results align with FTIR data, which showed complete NCO conversion in the BP-3 formulation, unlike the other compositions tested (Figure 14). Notably, the BP-3 formulation contained the highest BA bark biomass content (11.1%), making it the most advantageous in terms of balancing the biomass content with the compression properties of a material.

The composition based on liquefied extractives, abbreviated as BP-4, exhibited slightly lower characteristics but still showed improvements, particularly in rigidity, compared to the reference compositions, including the Ref. 2 composition, which contains trifunctional polyol Lupranol 3300 in combination with the hexafunctional polyol Lupranol 3422 (Table 3). The presence of a free NCO group peak in the FTIR spectrum of the BP-4 composition indicates that, unlike in oxypropylated extractives, some OH groups in the liquefied extractives remained inaccessible, decreasing NCO conversion and potentially resulting in reduced compression characteristics of this PUR foam. Mixing oxypropylated and liquefied extractives in equal proportions in the BP-5 formulation improved the compression properties compared to BP-4 (Figure 15).

The effect of bark biomass remaining after extraction, used as a PUR foam filler, on compression properties was studied using Ref. 3, BP-3, and BP-5 compositions (Table 4). Proper dispersion of the filler within the polyols, avoiding agglomerate formation, is essential for accurately assessing the filler’s effect on PUR foam properties [68]. In this study, filler amounts of 10–40 pbw per 100 pbw of polyol were introduced into the polyol mix of the Ref. 2 composition using a high-shear and high-speed stirrer, followed by PUR foam synthesis. The dependence of compression characteristics on the filler content was similar across both series of PUR foams obtained (Figure S7 in the Supplementary Materials). Consequently, a high-shear stirrer was used in subsequent cases.

A significant increase in the PUR foam apparent density, from approximately 40 to 52 kg∙m^−3^, was observed in the BP-3/F formulation, which contains oxypropylated extractives as a polyol, with the filler content increased up to 13.4%. This increase is likely due to the substantial retardation effect of the filler on the foam rise rate in the BP-3/F series, thereby lowering the foaming temperature and reducing the volume of the liberated gaseous phase (Figure 13d). For the BP-5/F series, the effect of the filler content on the apparent density was less pronounced, with density increasing from about 46 to 50 kg∙m^−3^. The reference system’s apparent density remained relatively consistent, ranging from 46 to 47 kg∙m^−3^. It is possible that traces of water, inevitably retained in the filler after drying, act as a chemical blowing agent, diminishing the impact of the filler content on foam density. Further study is needed to explain this effect accurately.

**Figure 16:**
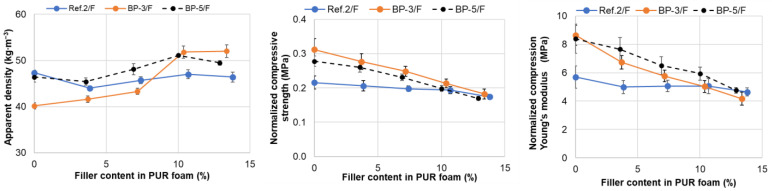
Apparent density, normalized strength, and Young’s modulus under axial compression parallel to the foaming direction of reference and bio-polyol-based rigid PUR foams as a function of the filler content (sample abbreviations are consistent with Table 3).

As the filler content in the foam compositions increased, both the compressive strength and modulus steadily decreased (Figure 16). This effect was more pronounced in the bio-polyol-based compositions. At a natural filler content of 12.9–13.9%, the normalized compressive strength across all PUR foam compositions was similar, approximately 0.17–0.18 MPa, in comparison to 0.31, 0.28, and 0.22 MPa for the BP-3, BP-5, and Ref. 2 compositions, respectively. This could be explained by a combination of factors, including the appearance of physical defects in the PUR matrix and a decrease in crosslinking, likely due to restricted NCO conversion in highly viscous compositions. Nevertheless, the most densely filled materials meet the CS(10/Y)150 level for compressive strength in rigid PUR foams, making them suitable for building insulation applications according to EN 14315-1 standards. In this case, up to 22–24% of the fossil resources in PUR foam compositions based on bio-polyols will be replaced by bark-derived biomass (Figure S6 and Table 4).

#### 3.5.5. The Morphological Structure of PUR Foams

The total heat transport through low-density PUR foam insulation results from a combination of heat transfer via the solid polymeric matrix conduction, conduction of gas within the foam cells, and radiation [69].

While the thermal conductivity of the PUR matrix and the radiation component remain relatively constant, the gas phase conductivity varies over time due to the diffusion of physical blowing agents and CO_2_ out of the cells. These agents are eventually replaced by air, which has a higher thermal conductivity [70].

For instance, the thermal conductivities of air, CO_2_, and hydrofluoro-olefin (Opteon ™) gases at 20 °C are 0.0260 W∙m^−1^∙K^−1^, 0.0168 W∙m^−1^∙K^−1^, and 0.0104 W∙m^−1^∙K^−1^, respectively [71].

Therefore, the closed cell content is an important parameter of the morphological structure of PUR foams in terms of slowing down the diffusion of blowing agents that reduce convective heat transfer [63,69].

**Figure 17:**
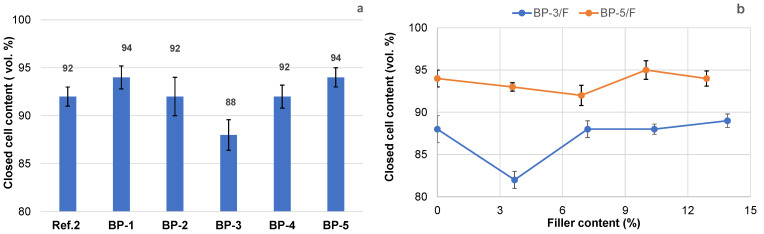
The closed-cell content in reference and bio-polyol based PUR foams (**a**); the effect of filler content on the closed-cell content in bio-polyol based foams (**b**) (sample abbreviations are consistent with Table 3 and Table 4).

According to EN 14315-1, the PUR foams under study, both reference and bio-based, can be characterized as closed-cell foams, as they contain over 90% closed cells (Figure 17a). A slightly lower closed-cell content was observed in the BP-3 composition, likely due to the high activity and low viscosity of the polyol system, which may have disrupted the balance between the rate of gas-phase formation and the rate of the polymer viscosity increase.

No negative effect of filler content on the closed-cell content in PUR foams, developed from a mixture of oxypropylated and liquified extractives, was observed (Figure 17b). For PUR foam based on oxypropylated extractives, a slight decrease in the closed-cell content was noted with a 3.5% filler; however, as the filler content increased, the closed-cell content remained similar to that of the unfilled sample. Although PUR foams based on oxypropylated extractives have a slightly lower closed-cell content, they still predominantly exhibit a closed-cell structure that meets the CCC3 level (80–89% closed cells) for building application standards according to EN 14315-1. Therefore, relatively low thermal conductivity can be expected in all bio-polyol-based PUR foams, including those with fillers.

**Figure 18:**
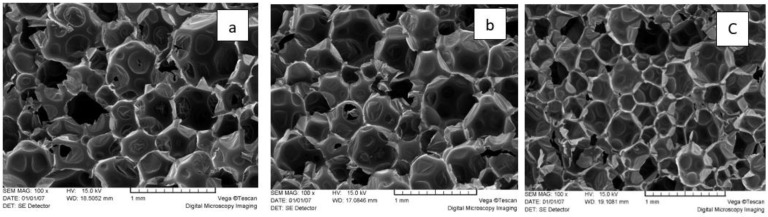
SEM images of reference and bio-polyols-based PUR foams in parallel with foaming directions. Ref. 2 (**a**); BP-3 (**b**); BP-4 (**c**).

Interestingly, the cell size of PUR foams based on liquified biomass (Figure 18c) was visibly smaller than that of the reference and oxypropylated extractive-based PUR foams (Figure 18a,b). Therefore, at comparable values of closed-cell content and apparent density, the thermal conductivity of foams using the BP-4 formulation could be lower than that of the other PUR foam compositions discussed above [63,69]). The measured values of the thermal conductivity coefficient of both reference (Ref. 2) and bio-polyol-based PUR foam (BP-5) were the same at 0.0225 W∙m^−1^∙K^−1^, conforming to their high thermal isolation properties. Testing of the thermal conductivity coefficient for other above-mentioned PUR foams, including filled compositions, is planned.

#### 3.5.6. Thermal Degradation of PUR Foams in Air

In oxidative environments, both thermal stress and the oxidative degradation of PUR chains overlap, accelerating the degradation of PUR materials compared to that in an inert atmosphere [72]. It is recognized that the thermal degradation of materials, accompanied by the release of gaseous products that support combustion, is the initial step in solid combustion processes [73]. Therefore, the thermal decomposition of PUR foam in oxidative media is a key characteristic influencing its suitability for specific practical applications.

**Figure 19:**
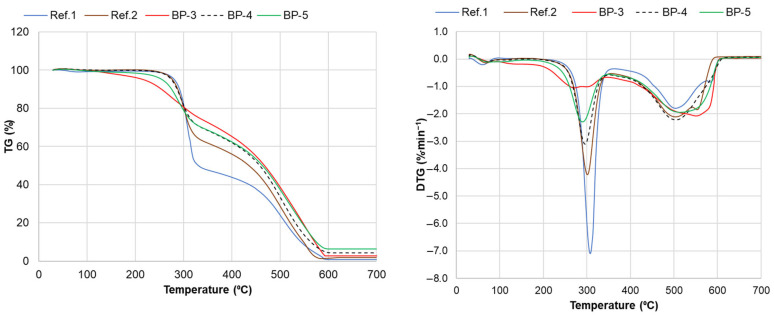
The TG and DTG curves of reference PUR foams and bio-polyol-based PUR foams in air.

Thermogravimetric analysis reveals two major stages in the degradation of PUR foams during non-isothermal heating in the air (Figure 19). The first stage, occurring between 200 and 360 °C with peak rates at 285–310 °C, is attributed to the degradation of aryl isocyanate-alkyl alcohol urethane bonds. This degradation produces gaseous or tar-like volatiles, including simple hydrocarbons, vinyl ethers, primary or secondary amines, isocyanates, and other oxidizable compounds that release heat at oxidation [72].

The second stage, at a higher temperature range of 360–600 °C with peak rates at 500–550 °C, involves complete thermal breakdown of the polymer chains. This process produces volatile compounds, including aliphatic alcohols with branched chains, acetonitrile, benzonitrile, and solid char [72,73]. These products are then oxidized under either homogeneous or heterogeneous conditions. 

The effect of bio-polyols on the thermogravimetric characteristics of their PUR foam-based compositions, compared to reference compositions, was rather complex [72].

The temperature at a 5% weight loss, defined as the onset temperature for degradation, was similar for PUR foams based on liquefied extractives (BP-4) and the reference compositions (Table 5). However, for the BP-5 system based on oxypropylated extractives, the T5% value was 217 °C, compared to 272 °C and 282 °C for the reference PUR foams (Table 5). The use of a blend of oxypropylated and liquefied biomass raised the degradation onset temperature of BP-5 to 256 °C. We propose that the presence of propylene diols in the oxypropylated extractives results in some PUR network fragments with a low crosslink density, thereby reducing their thermal stability [74].

It was established that the maximum-degradation-rate temperature of unfilled bio-polyol-based foams ranged from 285 to 295 °C, following the same order as T5% values: BP-3 < BP-5 < BP-4. The T_max_ values for the reference compositions were 15–20 °C higher, but the maximum degradation rate of bio-polyol-based foams was visibly lower and ranked as follows: BP-3 < BP-5 < BP-4 < Ref. 2 < Ref. 1. For instance, the maximum degradation rate for BP-3 systems containing oxypropylated extractives was 6.5 and 3.8 times lower than that of the reference PUR foam based on single-functional Lupranol 3300 and a blend with six-functional Lupranol 3422, respectively.

Bio-polyol-based PUR foams also demonstrated improved thermal characteristics in terms of the temperature at which 50% weight loss occurred and the residual weight remaining at 500 °C compared to both reference systems. The PUR foam based on oxypropylated extractives exhibited the highest T_50%_ and Δm_500 °C_ values (Table 5). Based on the above results, we conclude that incorporating bio-polyols into the PUR foam composition decreases weight loss in the volatile formation stage (200–350 °C) while increasing weight retention in the high-temperature range (450–600 °C), which is predominantly associated with char combustion. It is known that the flammability of PUR materials is linked to the release of flammable gaseous decomposition products during the initial degradation stage [72]. Therefore, the lower flammability of bio-polyol-based PUR foam versus reference could be awaited.

As shown earlier, the total biomass content in bio-polyol-based PUR foam matrices reached approximately 24% in the BP-3/F formulation and 22% in the BP-5/F formulation when about 14% extracted bark was added in them as a filler (Figure S6; Table 3). The effect of the filler on the starting temperature of foam degradation was found to be minimal. However, a significant reduction in thermal stability was observed for PUR foams based on both oxypropylated extractives and a mixture of liquefied and oxypropylated extractives, as indicated by decreases in the T_50%_ and Δm_500 °C_ values (Table 5). Additionally, the maximum degradation rate doubled in filled PUR foam based on oxypropylated extracts (Figure 19).

**Figure 20:**
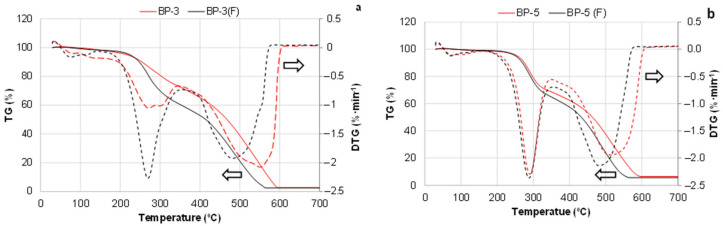
TG and DTG curves in air for unfilled (BP-3, BP-5) (**a**) and 13%-filler-containing (BP-3/F, BP-5/F) bio-polyol-based PUR foams (**b**).

For both filled compositions, the high-temperature peaks for char oxidation shifted 30–50 °C lower compared to the unfilled compositions. Additionally, complete combustion occurred at lower temperatures. This can be attributed to the lower activation energy of bark-derived char oxidation, which reached its highest rate at around 450 °C, compared to 500–550 °C observed for bio-polyol-based PUR foams (Figure 9a,b and Figure 20a,b). Consequently, the decreased thermal stability of the filled foams follows an additive principle, as the incorporation of a material with lower thermal stability than the PUR matrix reduces the overall stability. The lower crosslinking of the PUR matrix, due to increased diffusion restrictions preventing complete NCO conversion in filled compositions, may also contribute to their lower thermal stability compared to unfilled foams, similar to their compression properties. To assess the correlation between these thermogravimetric results and the flammability of the PUR foams, cone calorimeter combustion tests will be conducted as part of the continuation of this research.

The presented study demonstrated that polyols based on BA bark extractives are competitive with other lignocellulosic-biomass-derived polyols obtained through conventional oxypropylation in terms of the morphology and mechanical and thermal characteristics of synthesized rigid PUR foams. In contrast to conventional oxypropylation, two proposed methods of bark extractive liquefaction open promising prospects for up-scaling due to their low pressure and temperature requirements and the absence of organic solvents in the processing. Finally, the presented study has shown, as well, the rather complex dependence of PUR foam properties on the biomass content. Steady improvements in material characteristics with increasing biomass contents are not always achievable. A reasonable compromise must be found, considering the material properties that are critical for each potential application field.

## 4. Conclusions

Novel bio-polyols were synthesized through the acid-free liquefaction of BA bark extractives using the commercial polyether polyol Lupranol 3300 and “green” oxypropylation with propylene carbonate. Under optimal conditions, bio-polyols containing 21% and 27% biomass were obtained via the liquefaction and oxypropylation processes. These bio-polyols had OHV values of 539 and 464 mg KOH·g^−1^ and viscosities of 1050 and 7250 mPa·s at 25 °C, respectively. Unlike the oxypropylated extractives, which were enriched with uniform aliphatic OH functionality, the liquefied biomass contained both aliphatic and acidic OH groups, reducing polyol activity in PUR foam compositions.

The study demonstrated the highest activity in PUR foam compositions containing oxypropylated extractives as bio-polyols, with no unreacted isocyanate observed in the final material, unlike other compositions and reference samples. At an apparent density of 40 kg/m^3^, PUR foam based on oxypropylated extractives exhibited superior compression characteristics, with a strength of 0.31 MPa and Young’s modulus of 8.6 MPa, compared to 0.18–0.22 MPa and 4.9–5.7 MPa for the reference compositions. Additionally, the maximum thermal oxidative degradation rate of the bio-polyol-based foam was 3.8–6.5 times lower, while the residual weight at 500 °C was 1.2–1.6 times higher compared to the reference systems.

The composition based on liquefied extractives showed lower performance but still demonstrated improved strength and thermal stability compared to reference foams. By combining both bio-polyols, the foaming rise rate, along with the mechanical and thermal degradation properties of PUR foams, could be optimized while maintaining a biomass content of approximately 10%.

No significant effect on foam morphology was observed, with the closed-cell content in both reference and bio-polyol-based foams remaining at around 90%, resulting in a low thermal conductivity of 0.0225 W∙m^−1^·K^−1^ for both bark polyol-derived and reference foams.

Adding 3.7–13.9% extracted bark as a filler increased the renewable content in materials to 22–24%, with a decrease in the compression strength and thermal stability, while maintaining a similar closed-cell content to unfilled foams. Nevertheless, the densest filled materials met the CS (10/Y)150 compressive strength level for rigid PUR foams, making them suitable for building insulation applications according to EN 14315-1 standards.

The results indicate that black alder bark is a promising natural resource for bio-based PUR foam production.

## Figures and Tables

**Table 1:** The elemental composition and ash content in BA bark, extractives, and residual bark based on dry matter (DM).

Sample	Elemental Composition %				Ash Content (%)	Fixed Carbon Content ^2^ (%)
	C	H	N	O ^1^		
Un-treated bark	52.1 ± 0.6	5.98 ± 0.04	0.38 ± 0.04	38.4	3.1 ± 0.2	27.2 ± 0.5
Extractives	54.5 ± 0.3	5.76 ± 0.03	0.39 ± 0.01	38.2	1.2 ± 0.1	42.5 ± 1.0
Residual bark	51.8 ± 0.2	6.05 ± 0.02	0.34 ± 0.01	39.1	2.70 ± 0.02	28.0 ± 0.3

^1^—by difference; ^2^—the residual portion of biomass at 850 °C, as determined via TG analysis performed in argon corrected for the ash content.

**Table 2:** The composition and characteristics of a larger batch of bio-polyol synthesized via the reaction of BA bark extractives with PC in optimal conditions.

Composition ^1^ (%)			Characteristics of Bio-Polyol				
Extractives	PC	DBU	Biomass Content ^2^ (%)	DBU Content ^2^ (%)	OHV, mgKOH∙g^−1^	Viscosity at 25 °C (mPa∙s)	H_2_O by K.F., %
17.5	79.6	3.0	26.5	4.4	464	1050	0.09

^1^ Starting suspension; ^2^ calculated data.

**Table 3:** Compositions of references and bio-polyol-based rigid PU foams.

Position	Formulation						
	Ref. 1	Ref. 2	BP-1	BP-2	BP-3	BP-4	BP-5
Composition (pbW): Lupranol 3300 Lupranol 3422 Oxypropylated extractives Liquified extractives	100 - -	70 30 - -	- 30 70 -	- 30 - 70	- - 100 -	- - - 100	- - - 50 50
Water	0.50	0.50	0.50	0.45	0.50	0.35	0.45
Catalyst Polycat 5	0.7	0.7	-	0.7	-	-	-
Surfactant, Niax Silicone	1.5	1.5	1.5	1.5	1.5	1.5	1.5
Blowing agent Opteon ^TM^ 1100	20	20	20	20	20	20	20
pMDI	119.5	126.9	139.1	151.4	136.9	157.5	147.2
B/A ratio ^1^	0.97	1.03	1.14	1.23	1.12	1.28	1.21
Bark biomass content in PUR (%) ^2^	-	-	7.7	5.8	11.1	8.1	9.5

^1^ All formulation ingredients, except for pMDI, were defined as Component A, while pMDI was defined as Component B. ^2^ It was recalculated based on the PUR matrix by excluding the amount of the physical blowing agent from the calculation.

**Table 4:** Compositions of references and bio-polyol-based rigid PUR foams filled with extracted bark.

Position	Formulation		
	Ref. 2/F	BP-3/F	BP-5/F
Composition (pbW): Lupranol 3300 Lupranol 3422 Oxypropylated extract Liquified extract	70 30 - -	- - 100 -	- - - 50 50
Filler	10–40	10–40	10–40
Water	0.50	0.50	0.45
Catalyst Polycat 5	0.7	-	-
Surfactant, Niax Silicone	1.5	1.5	1.5
Blowing agent Opteon ^TM^ 1100	20	20	20
pMDI	126.9	136.9	147.2
B/A ratio ^1^	1.03	1.12	1.21
Biomass content in PUR (%) ^2^	4.2–13.8	14.9–24.2	13.1–22.1

^1^ All formulation ingredients, except for pMDI and filler, were defined as Component A, while pMDI was defined as Component B. ^2^ It was recalculated based on the PUR matrix by excluding the amount of the physical blowing agent from the calculation.

**Table 5:** The thermogravimetric parameters of the non-isothermal oxidative degradation of reference and bio-polyol-based PUR foams (filled and unfilled) and extracted bark used as a natural filler.

Sample	T_5%_ (°C) ^a^	${\frac{dm}{dt}}_{\max}$ (%∙min ^−1^) ^b^	T_max_ (°C) ^c^	T_50%_(%∙min^−1^) ^d^	Δm_500 °C_ (%) ^e^
Ref. 1	282 ± 5	7.1 ± 0.4	308 ± 8	350 ± 10	24.2 ± 1.0
Ref. 2	272 ± 6	4.2 ± 0.2	303 ± 2	436 ± 9	32.1 ± 1.2
BP-3	217 ± 5	1.1 ± 0.06	285 ± 3	468 ± 10	38.8 ± 2.0
BP-3/F	224 ± 2	2.3 ± 0.1	280 ± 5	414 ± 12	21.1 ± 1.6
BP-4	275 ± 3	3.1 ± 0.2	295 ± 6	458 ± 8	33.7 ± 2.5
BP-5	256 ± 4	2.3 ± 0.1	291 ± 4	463 ± 10	37.5 ± 2.8
BP-5/F	251 ± 5	2.4 ± 0.1	288 ± 3	432 ± 7	23.9 ± 1.1
Extracted bark (filler)	233 ± 6	2.5 ± 0.2	300 ± 2	357 ± 10	6.0 ± 1.0

^a^ the temperature when 5% weight loss was achieved; ^b^ the maximal rate of weight loss; ^c^ the temperature at which the maximal weight loss was observed; ^d^ the temperature when 50% weight loss was achieved; ^e^the residual weight at 500 °C.

## Data Availability

The data presented in this study are available on request from the corresponding authors. Main reason for selecting this data availability statement comes from requirements set by the funding institution. Funding provider (the Latvian Council of Science) does not require us to upload raw data to data repositories, so obtained raw data is saved privately by us and disseminated upon request.

## Supplementary Materials

The following supporting information can be downloaded at: https://www.mdpi.com/article/10.3390/ma18010050/s1, Figure S1: The TG/DTG curves of BA bark biomass: extractives, untreated, and extracted bark in argon; Table S1: Identification of the compounds present in water extracts of BA bark isolated using MW-assisted extraction methods using UHPLC-MS/MS; Figure S2: The structural formula of oregonin; Figure S3: The schematic view of oregonin oxypropylation with PC; Figure S4: The structural formula of 8-diazabicyclo [5.4.0]undec-7-ene (DBU); Figure S5: PUR foam samples: abbreviations are the same as in Table 3; Figure S6: The effect of filler content on the total portion of bark biomass in bio-polyol-based PUR foams; Figure S7: The effect of filler content on the compression characteristics of Ref. 2 PUR foam compositions prepared by using high shear and a high-rate stirrer.

## References

[1] Grand View Research Polyurethane Foam Market Size, Share & Trends Analysis Report by Product (Rigid Foam, Flexible Foam), By Application (Bedding & Furniture, Transportation, Packaging, Construction, Electronics, Footwear), by Region, and Segment Forecasts, 2024–2030. https://www.grandviewresearch.com/industry-analysis/polyurethane-foam-market.

[2] Vieira F.R., Magina S., Evtuguin D.V., Barros-Timmons A. (2022). Lignin as a Renewable Building Block for Sustainable Polyurethanes. Materials.

[3] Amran U.A., Salleh K.M., Zakaria S., Roslan R., Chia C.H., Jaafar S.N.S., Sajab M.S., Mostapha M. (2021). Production of Rigid Polyurethane Foams Using Polyol from Liquefied Oil Palm Biomass: Variation of Isocyanate Indexes. Polymers.

[4] Hirvonen J., Jokisalo J., Heljo J., Kosonen R. (2019). Towards the EU Emission Targets of 2050: Cost-Effective Emission Reduction in Finnish Detached Houses. Energies.

[5] Mordor Intelligence Methylene Diphenyl Di-Isocyanate (MDI) Market Size. https://www.mordorintelligence.com/industry-reports/methylene-diphenyl-di-isocyanate-mdi-market.

[6] Kreye O., Mutlu H., Meier M.A.R. (2013). Sustainable Routes to Polyurethane Precursors. Green Chem..

[7] Rubens M., Van Wesemael M., Feghali E., Lufungula L.L., Blockhuys F., Vanbroekhoven K., Eevers W., Vendamme R. (2022). Exploring the Reactivity of Aliphatic and Phenolic Hydroxyl Groups in Lignin Hydrogenolysis Oil towards Urethane Bond Formation. Ind. Crops Prod..

[8] Kaikade D.S., Sabnis A.S. (2023). Polyurethane Foams from Vegetable Oil-Based Polyols: A Review. Polym. Bull..

[9] Lavazza J., Zhang Q., de Kergariou C., Comandini G., Briscoe W.H., Rowlandson J.L., Panzera T.H., Scarpa F. (2024). Rigid Polyurethane Foams from Commercial Castor Oil Resins. Polym. Test.

[10] Tan S., Abraham T., Ference D., Macosko C.W. (2011). Rigid Polyurethane Foams from a Soybean Oil-Based Polyol. Polymer.

[11] Członka S., Bertino M.F., Kośny J., Strąkowska A., Masłowski M., Strzelec K. (2018). Linseed Oil as a Natural Modifier of Rigid Polyurethane Foams. Ind. Crops Prod..

[12] Coman A.E., Peyrton J., Hubca G., Sarbu A., Gabor A.R., Nicolae C.A., Iordache T.V., Averous L. (2021). Synthesis and Characterization of Renewable Polyurethane Foams Using Different Biobased Polyols from Olive Oil. Eur. Polym. J..

[13] Kirpluks M., Kalnbunde D., Walterova Z., Cabulis U. (2017). Rapeseed Oil as Feedstock for High Functionality Polyol Synthesis. J. Renew. Mater..

[14] Asare M.A., Kote P., Chaudhary S., de Souza F.M., Gupta R.K. (2022). Sunflower Oil as a Renewable Resource for Polyurethane Foams: Effects of Flame-Retardants. Polymers.

[15] Valero M.F., Gonzalez A. (2012). Polyurethane Adhesive System from Castor Oil Modified by a Transesterification Reaction. J. Elastomers Plast..

[16] Malewska E., Polaczek K., Kurańska M. (2022). Impact of Various Catalysts on Transesterification of Used Cooking Oil and Foaming Processes of Polyurethane Systems. Materials.

[17] Yakushin V., Misane M., Bikovens O., Vilsone D., Sevastyanova I. (2016). Synthesis of Trimethylolpropane Esters of Tall Oil Fatty Acids and Properties of Polyurethane Coatings on Their Basis. J. Coat. Technol. Res..

[18] Kauliņa E., Abolins A., Fridrihsone A., Kirpluks M. (2022). Potential of Crude Tall Oil Polyols for Rigid Polyurethane Foam Production and Their Comparison with Tall Oil Fatty Acids Polyols. Mater. Sci. Forum.

[19] Kirpluks M., Vanags E., Abolins A., Michalowski S., Fridrihsone A., Cabulis U. (2020). High Functionality Bio-Polyols from Tall Oil and Rigid Polyurethane Foams Formulated Solely Using Bio-Polyols. Materials.

[20] Phung Hai T.A., Tessman M., Neelakantan N., Samoylov A.A., Ito Y., Rajput B.S., Pourahmady N., Burkart M.D. (2021). Renewable Polyurethanes from Sustainable Biological Precursors. Biomacromolecules.

[21] Członka S., Strąkowska A., Kairytė A. (2020). Application of Walnut Shells-Derived Biopolyol in the Synthesis of Rigid Polyurethane Foams. Materials.

[22] Bontaş M.G., Diacon A., Călinescu I., Rusen E. (2023). Lignocellulose Biomass Liquefaction: Process and Applications Development as Polyurethane Foams. Polymers.

[23] Oliveira W.D., Glasser W.G. (1989). Engineering Plastics from Lignin. XVI. Starlike Macromers with Propylene Oxide. J. Appl. Polym. Sci..

[24] Kelley S.S., Glasser W.G., Ward T.C. (1988). Engineering Plastics from Lignin XIV. Characterization of Chain-Extended Hydroxypropyl Lignins. J. Wood Chem. Technol..

[25] Mahmood N., Yuan Z., Schmidt J., Xu C. (2016). Depolymerization of Lignins and Their Applications for the Preparation of Polyols and Rigid Polyurethane Foams: A Review. Renew. Sustain. Energy Rev..

[26] D’Souza J., George B., Camargo R., Yan N. (2015). Synthesis and Characterization of Bio-Polyols through the Oxypropylation of Bark and Alkaline Extracts of Bark. Ind. Crops Prod..

[27] Evtiouguina M., Barros-Timmons A., Cruz-Pinto J.J., Neto C.P., Belgacem M.N., Gandini A. (2002). Oxypropylation of Cork and the Use of the Ensuing Polyols in Polyurethane Formulations. Biomacromolecules.

[28] Serrano L., Alriols M.G., Briones R., Mondragón I., Labidi J. (2010). Oxypropylation of Rapeseed Cake Residue Generated in the Biodiesel Production Process. Ind. Eng. Chem. Res..

[29] Pavier C., Gandini A. (2000). Oxypropylation of Sugar Beet Pulp. Ind. Crops Prod..

[30] Gandini A., Belgacem M.N. (2008). Partial or Total Oxypropylation of Natural Polymers and the Use of the Ensuing Materials as Composites or Polyol Macromonomers. Monomers, Polymers and Composites from Renewable Resources.

[31] Vieira F.R., Gama N.V., Evtuguin D.V., Amorim C.O., Amaral V.S., Pinto P.C.O.R., Barros-Timmons A. (2023). Bio-Based Polyurethane Foams from Kraft Lignin with Improved Fire Resistance. Polymers.

[32] Kühnel I., Saake B., Lehnen R. (2017). Comparison of Different Cyclic Organic Carbonates in the Oxyalkylation of Various Types of Lignin. React. Funct. Polym..

[33] Arshanitsa A., Pals M., Godina D., Bikovens O. (2024). The Oxyalkylation of Hydrophilic Black Alder Bark Extractives with Propylene Carbonate with a Focus on Green Polyols Synthesis. J. Renew. Mater..

[34] Yip J., Chen M., Szeto Y.S., Yan S. (2009). Comparative Study of Liquefaction Process and Liquefied Products from Bamboo Using Different Organic Solvents. Bioresour. Technol..

[35] Serrano L., Rincón E., García A., Rodríguez J., Briones R. (2020). Bio-Degradable Polyurethane Foams Produced by Liquefied Polyol from Wheat Straw Biomass. Polymers.

[36] Jin Y., Ruan X., Cheng X., Lü Q. (2011). Liquefaction of Lignin by Polyethyleneglycol and Glycerol. Bioresour. Technol..

[37] D’Souza J., Camargo R., Yan N. (2014). Polyurethane Foams Made from Liquefied Bark-based Polyols. J. Appl. Polym. Sci..

[38] D’Souza J., Wong S.Z., Camargo R., Yan N. (2016). Solvolytic Liquefaction of Bark: Understanding the Role of Polyhydric Alcohols and Organic Solvents on Polyol Characteristics. ACS Sustain. Chem. Eng..

[39] Cinelli P., Anguillesi I., Lazzeri A. (2013). Green Synthesis of Flexible Polyurethane Foams from Liquefied Lignin. Eur. Polym. J..

[40] Zhang J., Du M., Hu L. (2016). Factors Influencing Polyol Liquefaction of Nut Shells of Different Camellia Species. Bioresources.

[41] Pásztory Z., Mohácsiné I.R., Gorbacheva G., Börcsök Z. (2016). The Utilization of Tree Bark. Bioresources.

[42] Jeżo A., Wronka A., Dębiński A., Kristak L., Reh R., Rizhikovs J., Kowaluk G. (2023). Influence of Upcycled Post-Treatment Bark Biomass Addition to the Binder on Produced Plywood Properties. Forests.

[43] Salca E.-A. (2019). Black Alder (*Alnus Glutinosa* L.)—A Resource for Value-Added Products in Furniture Industry Under European Screening. Curr. For. Rep..

[44] Arshanitsa A., Ponomarenko J., Lauberte L., Jurkjane V., Pals M., Akishin Y., Lauberts M., Jashina L., Bikovens O., Telysheva G. (2022). Advantages of MW-Assisted Water Extraction, Combined with Steam Explosion, of Black Alder Bark in Terms of Isolating Valuable Compounds and Energy Efficiency. Ind. Crops Prod..

[45] Pals M., Lauberte L., Ponomarenko J., Lauberts M., Arshanitsa A. (2022). Microwave-Assisted Water Extraction of Aspen (*Populus Tremula*) and Pine (*Pinus sylvestris* L.) Barks as a Tool for Their Valorization. Plants.

[46] Argyropoulos D. (1994). Quantitative Phosphorus-31 NMR Analysis of Lignins, a New Tool for the Lignin Chemist. J. Wood Chem. Technol..

[47] Argyropoulos D.S., Pajer N., Crestini C. (2021). Quantitative 31P NMR Analysis of Lignins and Tannins. J. Vis. Exp..

[48] Gosz K., Kowalkowska-Zedler D., Haponiuk J., Piszczyk Ł. (2020). Liquefaction of Alder Wood as the Source of Renewable and Sustainable Polyols for Preparation of Polyurethane Resins. Wood Sci. Technol..

[49] Zakis G.F. (1994). Functional Analysis of Lignins and Their Derivatives.

[50] Ionescu M. (2005). Chemistry and Technology of Polyols for Polyurethanes.

[51] Olszewski A., Kosmela P., Vēvere L., Kirpluks M., Cabulis U., Piszczyk Ł. (2024). Effect of Bio-Polyol Molecular Weight on the Structure and Properties of Polyurethane-Polyisocyanurate (PUR-PIR) Foams. Sci. Rep..

[52] Arshanitsa A., Ponomarenko J., Pals M., Jashina L. (2023). Controlling the Reactivity of Hydrophilic Bark Extractives as Biopolyols in Urethane-Formation Reactions Using Various Catalysts. Ind. Crops Prod..

[53] Arshanitsa A., Ponomarenko J., Pals M., Jashina L., Lauberts M. (2023). Impact of Bark-Sourced Building Blocks as Substitutes for Fossil-Derived Polyols on the Structural, Thermal, and Mechanical Properties of Polyurethane Networks. Polymers.

[54] Alma M.H., Shiraishi N. (1998). Preparation of Polyurethane-like Foams from NaOH-Catalyzed Liquefied Wood. Holz Roh-Und Werkst..

[55] Robyt J.F. (1998). Essentials of Carbohydrate Chemistry.

[56] Zhang Z., Huber G.W. (2018). Catalytic Oxidation of Carbohydrates into Organic Acids and Furan Chemicals. Chem. Soc. Rev..

[57] Hellwig P. (2015). Infrared Spectroscopic Markers of Quinones in Proteins from the Respiratory Chain. Biochim. Biophys. Acta (BBA) Bioenerg..

[58] Kutyrev A.A. (1991). Nucleophilic Reactions of Quinones. Tetrahedron.

[59] Saunders J.H., Frisch K.C. (1962). Polyurethanes: Chemistry and Technology.

[60] Dulyanska Y., Cruz-Lopes L., Esteves B., Guiné R., Domingos I. (2024). FTIR Monitoring of Polyurethane Foams Derived from Acid-Liquefied and Base-Liquefied Polyols. Polymers.

[61] Sheridan J.E., Haines C.A. (1971). Qualitative and Quantitative Analyses of Isocyanurate-Containing Foams by Thermal Techniques. J. Cell. Plast..

[62] Reignier J., Méchin F., Sarbu A. (2021). Chemical Gradients in PIR Foams as Probed by ATR-FTIR Analysis and Consequences on Fire Resistance. Polym. Test.

[63] Ivdre A., Abolins A., Volkovs N., Vevere L., Paze A., Makars R., Godina D., Rizikovs J. (2023). Rigid Polyurethane Foams as Thermal Insulation Material from Novel Suberinic Acid-Based Polyols. Polymers.

[64] Park D.H., Park G.P., Kim S.H., Kim W.N. (2013). Effects of Isocyanate Index and Environmentally-Friendly Blowing Agents on the Morphological, Mechanical, and Thermal Insulating Properties of Polyisocyanurate-Polyurethane Foams. Macromol. Res..

[65] Hawkins M.C., O’Toole B., Jackovich D. (2005). Cell Morphology and Mechanical Properties of Rigid Polyurethane Foam. J. Cell. Plast..

[66] Shivakumar N.D., Deb A. (2022). Dependence of the Mechanical Properties of Rigid PU Foam on Density. J. Reinf. Plast. Compos..

[67] Sture B., Vevere L., Kirpluks M., Godina D., Fridrihsone A., Cabulis U. (2021). Polyurethane Foam Composites Reinforced with Renewable Fillers for Cryogenic Insulation. Polymers.

[68] Husainie S.M., Deng X., Ghalia M.A., Robinson J., Naguib H.E. (2021). Natural Fillers as Reinforcement for Closed-Molded Polyurethane Foam Plaques: Mechanical, Morphological, and Thermal Properties. Mater. Today Commun..

[69] Soloveva O.V., Solovev S.A., Vankov Y.V., Shakurova R.Z. (2022). Experimental Studies of the Effective Thermal Conductivity of Polyurethane Foams with Different Morphologies. Processes.

[70] Glicksman L., Stewart J. (1997). The Measurement of the Morphology of Closed Cell Foams Which Control the Overall Thermal Conductivity. Insulation Materials: Testing and Applications.

[71] Lemmon E.W., Jacobsen R.T. (2004). Viscosity and Thermal Conductivity Equations for Nitrogen, Oxygen, Argon, and Air. Int. J. Thermophys..

[72] Chattopadhyay D.K., Webster D.C. (2009). Thermal Stability and Flame Retardancy of Polyurethanes. Prog. Polym. Sci..

[73] Jiao L., Xiao H., Wang Q., Sun J. (2013). Thermal Degradation Characteristics of Rigid Polyurethane Foam and the Volatile Products Analysis with TG-FTIR-MS. Polym. Degrad. Stab..

[74] Gupta T., Adhikari B. (2003). Thermal Degradation and Stability of HTPB-Based Polyurethane and Polyurethaneureas. Thermochim. Acta.

