# Supplementary material for: The Complex Valorization of Black Alder Bark Biomass in Compositions of Rigid Polyurethane Foam
Source: Materials (Basel). 2024 Dec 26;18(1):50. doi: 10.3390/ma18010050 (PMC11721132; doi:10.3390/ma18010050)
Supplement: Supplementary file 1 [file materials-18-00050-s001.zip › materials-3365390-supplementary.pdf]

The Complex Valorization of Black Alder Bark Biomass in Compositions of Rigid Polyurethane Foam

Alexandr Arshanitsa *, Matiss Pals, Laima Vevere, Lilija Jashina and Oskars Bikovens

Latvian State Institute of Wood Chemistry, Dzerbenes Street 27, LV-1006 Riga, Latvia; matiss.pals@kki.lv (M.P.); laima.vevere@kki.lv (L.V.); lilija_jasina@inbox.lv (L.J.); oskars.bikovens@kki.lv (O.B.)
* Correspondence: alexandr.arsanica@kki.lv

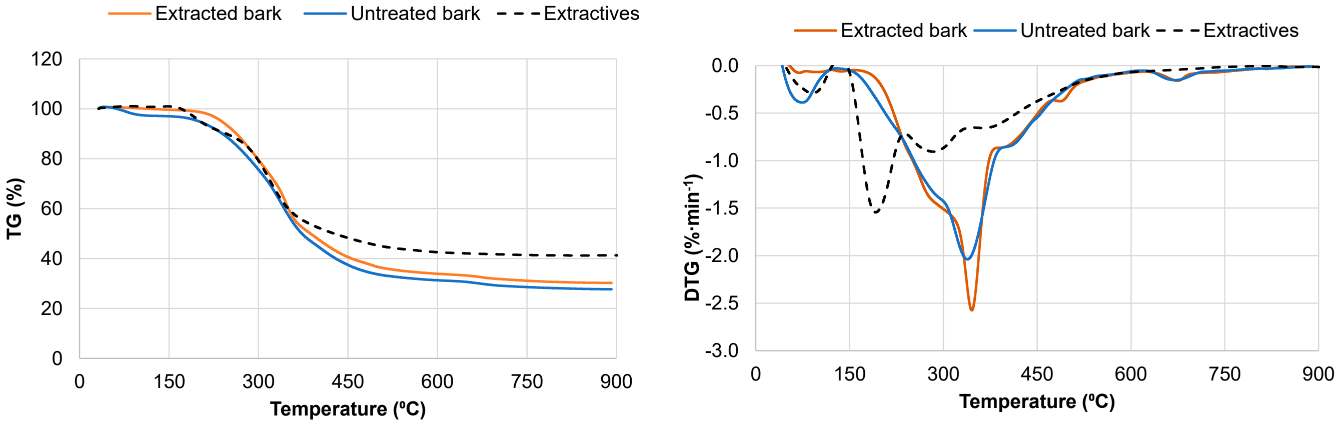

Figure S1. The TG/DTG curves of BA bark biomass: extractives, untreated and extracted bark in argon.

Table S1. Identification of the compounds present in water extracts of BA bark isolated MW-assisted extraction methods using UHPLC-MS/MS [46]

Peak	Ret. time, min	$(M\text{-}H)^-$ (m/z)	Fragments (m/z)	Tentative identification	Chemical group of compounds
1	0.39	341.1093	683; 1025	3-glucopyranosyloxy-1-(4-hydroxyphenyl)-butanone	Phenylbutanones
2	0.40	179.0565	305; 193; 59	The signals at m/z 179. 163 and 59 proved the presence of a glucosyl group (glucose)	Carbohydrates
3	0.46	191.0196	173; 111; 85	Quinic acid	Organic acids
4	1.93/2.04	337.0940	163; 191; 675	Coumaroylquinic acid	
9	2.78/2.99	507.1870	327; 205; 121; 1015	Hirsutenone hexoside	
10	3.04/3.28	477.1773	327; 205; 955	Oregonin	
11	3.43	1155.7119	477; 327; 205; 955 723;714; 609	Oregonin derivative	
12	3.44/3.73	345.1362	165; 691	Hirsutanonol	
13	3.69/4.02	461.1783	311;189	Aceroside VII	
14	3.77/4.11	461.1777	311; 205	1- (4-hydroxyphenyl) - 7- (3,4-dihydroxyphenyl) heptan-3-one-5-O-pentoside	
15	3.94/4.31	625.2496	493; 373; 331; 313	Rubranol C	
16	4.36/4.77	493.2060	331; 313; 987	Rubranoside A	
17	5.00/5.47	463.1997	331; 313; 295	Rubranol xyloside	
18	5.60/6.11	653.2368	311; 147	5(S)-1,7-di(3,4-dihydroxyphenyl)-5-O-b-D-(6-(E-p-coumaroylglucopyranosyl)) heptane-3-one	Diarylheptanoids (DAH)
19	6.32/6.48	327.1165	205; 121	Hirsutenone	
20	6.55/6.61	697.2511	327; 205; 121	5-1.7-di(3,4-dihydroxyphenyl)-5-O-β-d-(6-(3,4-dimethoxycinnamoyl glucopyranosyl)) heptane-3-one	
21	6.68	683.2715	637	1,7-di(3,4-dihydroxyphenyl)-5-O- -D-[6-(-3,4-dimethoxycinnamoylglucopyranosyl)]heptane	

22	7.27	293.1675	275; 193; 99	Gingerol-6	
6	2.41	577.1357	287; 305; 409; 417; 425; 433	Procyanidin B dimer	Condensed tannins
7	2.34	865.1998	577; 575; 289	Procyanidin B trimer	
8	2.39	1153.2666	865; 577; 289	Procyanidin B tetramer	
5	2.26	289.0720	245; 205	Catechin	Flavonoids

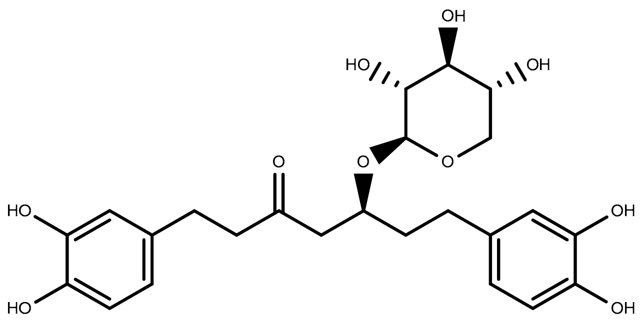

Figure S2. The structural formula of oregonin

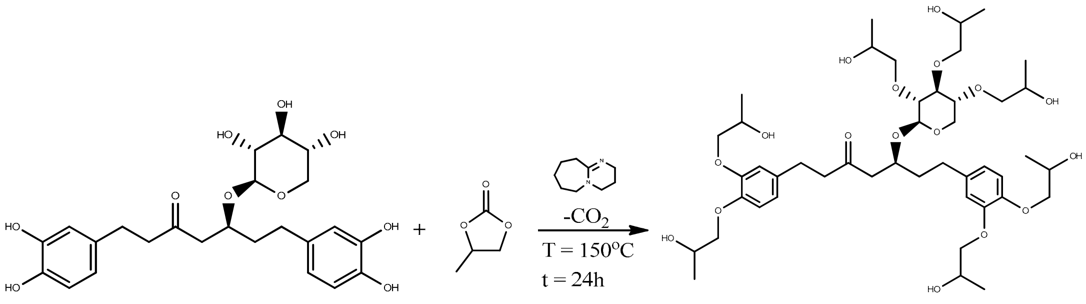

Figure S3. The schematic view of oregonin oxypropylation with PC

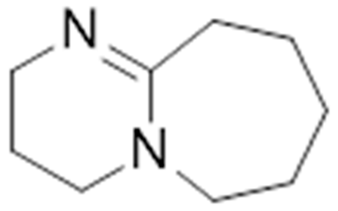

Figure S4. The structural formula of 8-diazabicyclo [5.4.0]undec-7-ene (DBU)

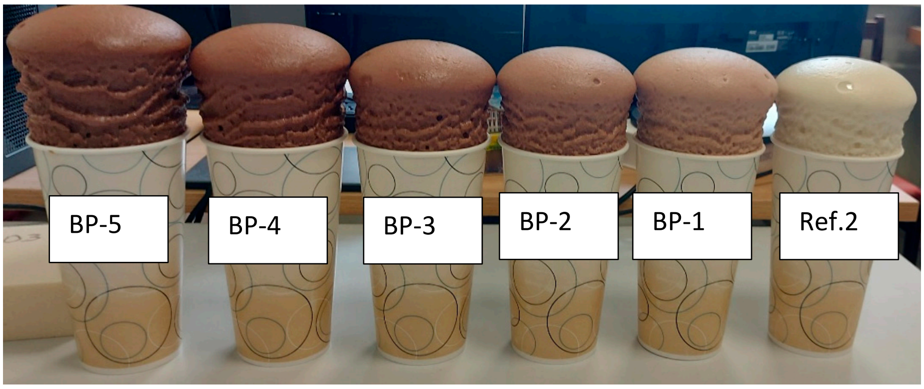

Figure S5. PUR foams samples: abbreviation the same as in Table 3.

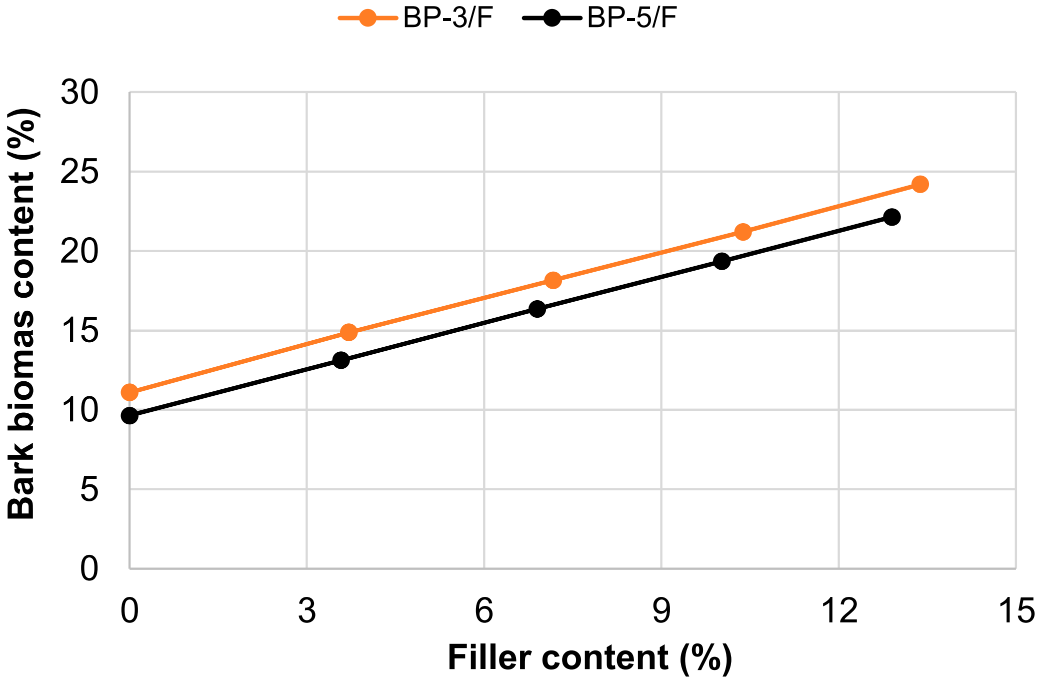

Figure S6. The effect of filler content on total portion of bark biomass in bio-polyol based PUR foams.

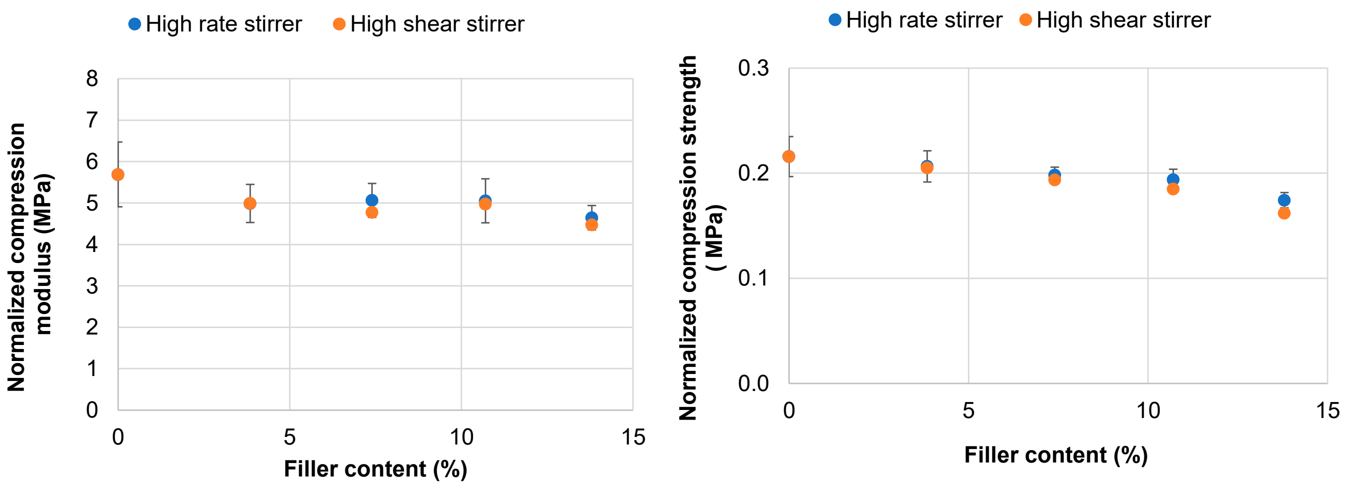

Figure S7. The effect of filler content on compression characteristics of Ref. 2 PUR foam compositions prepared by using high shear and a high-rate stirrers.
